# Multifunctional Mesoporous Titanium Dioxide Nanodrug for Corneal Haze Treatment and Its Mechanism

**DOI:** 10.34133/bmr.0202

**Published:** 2025-05-14

**Authors:** Tao Li, Xiaoli Wu, Yi Huang, Juan Tang, Yu Zhang, Yangrui Du, Zhiyu Du

**Affiliations:** ^1^ Department of Ophthalmology, The Second Affiliated Hospital of Chongqing Medical University, Chongqing 400010, China.; ^2^ Chongqing Key Laboratory of Ultrasound Molecular Imaging, The Second Affiliated Hospital of Chongqing Medical University, Chongqing 400010, China.; ^3^ Department of Ophthalmology, Ziyang Central Hospital, Ziyang, Sichuan 641300, China.; ^4^ Department of Endocrinology, Ziyang Central Hospital, Ziyang, Sichuan 641300, China.

## Abstract

Preventing and treating corneal haze is essential after corneal surface refractive surgery. However, the high intraocular pressure that results after applying traditional anti-inflammatory corticosteroids has attracted great attention. Therefore, we synthesized a multifunctional nanomedicine (Tet@TiO_2_) with controlled drug release, inflammation targeting, and good biocompatibility for corneal haze treatment. In this study, we discovered that Tet@TiO_2_ and tetrandrine (Tet), but not TiO_2_, displayed a characteristic absorption peak at 282 nm. Three weeks after transepithelial photorefractive keratectomy surgery, the Tet@TiO_2_ group displayed significant decreases in nuclear volume, corneal cell edema, type I and III collagen fiber expression, normal organelle morphology, and collagen fiber arrangement. Compared with those in the control and TiO_2_ groups, the α-smooth muscle actin, connective tissue growth factor, and type III collagen fibers in the Tet@TiO_2_ group decreased more significantly after fluorometholone eye drop and Tet treatment, indicating that Tet@TiO_2_ can effectively inhibit the expression of these inflammatory factors during corneal haze formation. Moreover, Tet@TiO_2_ showed good, sustained antibacterial properties. More importantly, we found that Tet@TiO_2_ could effectively down-regulate the expression of phosphatidylinositol 3-kinase (PI3K), protein kinase B (AKT), and B-cell lymphoma-2 (Bcl-2) and up-regulate the expression of Bcl-2-associated X protein (Bax) by modulating the inflammatory PI3K–AKT–Bax/Bcl-2 signaling pathway after corneal surface refractive surgery to effectively prevent and treat corneal haze by reducing the expression of inflammatory factors.

## Introduction

The complex healing process of corneal haze, which affects vision after refractive corneal surgery, occurs at the interface of the corneal epithelium and stroma in the surgical area [1–3]. The histopathological features of corneal haze include the activation and proliferation of corneal fibroblasts in the stromal layer, the accumulation of a large amount of extracellular matrix (ECM), and the disordered arrangement of coarse collagen fibers during postoperative wound healing [4,5]. Wilson [6] reported that after photorefractive keratectomy, LASIK, and femtosecond laser small incision corneal stromal lens extraction surgery (SMILE) procedures, many fibroblasts were present in the corneal stromal layer, and the cells had a large volume and nuclei and were rich in rough endoplasmic reticula, mitochondria, Golgi apparatuses, and other organelles, indicating high metabolism and vigorous proliferation, which easily led to the development of corneal haze. Jeon et al. [7] reported that the increased expression of transforming growth factor-β_2_ (TGF-β_2_) in corneal tissue after refractive corneal surgery induced the proliferation of corneal fibroblasts and their transformation into myofibroblasts, thus increasing the expression of α-smooth muscle actin (α-SMA), connective tissue growth factor (CTGF), and type III collagen, which are key factors in corneal haze development. Therefore, timely and regular corneal haze treatment is essential for patients that underwent refractive corneal surgery [8,9]. At present, corticosteroid drugs are the main method by which corneal haze is prevented and treated. Related studies have shown that CTGF plays an important regulatory role during tissue wound repair and is key in regulating TGF-β_2_. Corticosteroid drugs effectively reduce the expression of TGF-β_2_, thus decreasing CTGF synthesis in corneal fibroblasts to prevent and treat corneal haze [10]. However, research on the treatment of corneal haze with corticosteroids has focused on evaluating the outcomes of clinical treatment, such as improving the visual quality of patients after surgery and eliminating haze, and there is currently a lack of research on the mechanism of drug action. Additionally, the inappropriate use of corticosteroids can easily lead to complications, such as high intraocular pressure and cataracts, which limit their clinical use [11,12]. Therefore, it is very important to develop a new drug aimed at blocking the development of corneal haze with fewer side effects.

Nanomedicines that are easily modified and have controlled release and targeting properties and good biocompatibility have been applied to address the clinical drawbacks of corticosteroid treatment regimens [13–15]. Mesoporous titanium dioxide (TiO_2_) nanocarriers have been widely used during the manufacture of ophthalmic prostheses and artificial corneas because of their good corrosion resistance and antibacterial properties, suggesting that TiO_2_ has excellent potential as a nanodrug carrier in the field of ophthalmology [16,17]. Overall, the complete biodegradation, antimicrobial activity, relative nontoxicity, targeting, and controlled release performance of TiO_2_ have aroused our interest.

Tetrandrine (6,6′,7,12-tetramethoxy-2,2′-dimethyl-berbaman, Tet), a bis-benzylisoquinoline alkaloid isolated from the Chinese medicinal herb *Stephania tetrandra*, was initially used for the treatment of pulmonary fibrosis [18–20]. However, further studies have suggested that Tet also has antioxidant properties, as it promotes apoptosis and inhibits inflammatory reactions [21–24]. In addition, the therapeutic effects of Tet on ocular diseases such as dry eye, glaucoma, corneal haze, conjunctivitis, and uveitis have been researched [25–29]. Our previous studies suggested that Tet could inhibit the development of corneal haze in rabbits after Epi-LASIK surgery, possibly by modulating the TGF-β_2_–collagen III pathway [27]. Pharmacokinetic studies on Tet showed that after the application of a single eye drop, the highest concentration of Tet was found in corneal tissue with an elimination half-life of 115 to 140 min and an elimination rate constant of 0.06/min. Moreover, the calculated area under the curve in the cornea was 2,336.294 μg/(g·min), which is much higher than the areas under the curve in other intraocular tissues, such as the aqueous humor (approximately 8-fold), the iris ciliary body (5-fold), the lens (15-fold), and the vitreous body (25-fold). These results indicated that most of the drug entering the eye accumulated in the corneal tissue and that relatively small amounts of the drug entered other tissues in the eye. Our team speculated that the mechanism of this process may be related to the reduction in the expression of type III collagen and TGF-β_2_ and an imbalance in the ratio of Bcl-2-associated X protein (Bax)/B-cell lymphoma-2 (Bcl-2) expression, among other factors. Moreover, our research revealed that in addition to its an anti-inflammatory effect on ocular surfaces, Tet also lowered the intraocular pressure slightly; this observation is contrary to that after the long-term use of glucocorticoid drugs, which may lead to elevated intraocular pressure [25]. However, there is still a lack of research on the mechanism by which Tet affects corneal haze after refractive corneal surgery. Many studies investigating the treatment of pulmonary fibrosis with Tet have revealed that the pathological characteristics of pulmonary fibrosis are similar to those of development of corneal haze, such as the abnormal accumulation of ECM and α-SMA in myofibroblasts [30,31]. In addition, many studies have shown that Tet can down-regulate TGF-β_2_ by suppressing the phosphatidylinositol 3-kinase (PI3K)–protein kinase B (AKT)–mTOR signaling pathway to promote myofibroblast apoptosis. This leads to reductions in the levels of inflammatory factors related to the development of pulmonary fibrosis, such as α-SMA, CTGF, and type III collagen, to alleviate pulmonary fibrosis [32–35]. In summary, our research team suggested that Tet can treat corneal haze by regulating PI3K–AKT–Bax/Bcl-2 signaling after refractive corneal surgery. Therefore, the purpose of this study was to create a new, multifunctional nanomedicine with anti-inflammatory, antibacterial, and targeting effects that promotes slow drug release and displays low toxicity by encapsulating Tet in TiO_2_ and to further exploring the mechanism by which this drug prevents the development of corneal haze after refractive corneal surgery.

## Materials and Methods

### Synthesis of TiO_2_

TiO_2_ nanoparticles were synthesized via the sol–gel method. Tetrabutyl titanate was added to 20 ml of anhydrous ethanol with stirring for 10 min to obtain solution A. Moreover, 6 ml of glacial acetic acid and 1.5 ml of ultrapure water were added to 20 ml of anhydrous ethanol to obtain solution B. Then, solution A was slowly added dropwise to solution B, and the mixture was stirred for 30 min and heated to 60 °C for 24 h to obtain a transparent solution (sol). The obtained sol was further heated at 80 °C for 24 h to obtain a light yellow solid product. Finally, the product was calcined at 400 °C for 3 h to remove impurities, yielding TiO_2_ nanoparticles as a white powder.

### Synthesis of Tet@TiO_2_

First, 100 mg of mesoporous TiO_2_ nanoparticles, 60 mg of Tet, and 4 mg of lysine were mixed with 50 ml of methanol, after which the solvent was evaporated by rotary evaporation (50 °C, 120 rpm). Tet was subsequently loaded into the mesopores of the titanium dioxide nanoparticles to produce Tet@TiO_2_. After rotary evaporation to remove the solvent, the residue was dissolved in 50 ml of phosphate-buffered saline (PBS). After ultrasonication to fabricate a uniform dispersion, the mixture was subjected to cryogenic centrifugation at 12,000 rpm for 10 min. Then, the supernatant was centrifuged again under the same conditions to obtain the sample. Finally, the concentration of Tet@TiO_2_ in the sample was adjusted to the appropriate experimental concentration.

### Materials and characterization

The absorbance spectra of free Tet, TiO_2_, and Tet@TiO_2_ were acquired with an ultraviolet (UV) spectrophotometer (UV2600, Shimadzu, Japan) at room temperature. Transmission electron microscopy (TEM) was employed to view the morphology of TiO_2_ and Tet@TiO_2_. Furthermore, the particle size and zeta potential were measured using a laser particle size analyzer system (Nano ZS90, Malvern Instruments Ltd.). The average sizes of TiO_2_ and Tet@TiO_2_ were measured after 1, 2, 3, 4, 5, 6, and 7 d to evaluate the stability of the nanomaterials. The encapsulation efficiency (EE) and drug loading capacity (LC) were calculated using the following equations:

$$\begin{array}{c} \mathrm{EE}\;\left(\%\right)=\\ \text{amount of encapsulated drug}/\text{total amount of drug}\times 100\%, \end{array}$$ (1)

$$\begin{array}{c} \mathrm{LC}\;\left(\%\right)=\\ \text{amount of encapsulated drug}/\text{total weight of}\;{\mathrm{TiO}}_{2}\times 100\%, \end{array}$$ (2)

and

$$\begin{array}{c} \text{Amount of encapsulated drug}=\\ \text{total drug}-\text{free drug in the supernatant}. \end{array}$$ (3)

The Tet release performance was examined by high-performance liquid chromatography (VP-ODS 150 mm × 4.6 mm; flow rate: 1 ml/min; mobile phase: methyl alcohol:water:triethylamine, 750:250:0.5) after incubation at 4 °C (storage temperature), 25 °C (room temperature), and 33 °C (corneal temperature).

### Experimental animals

The animal care and experimental procedures complied with the Principles of Laboratory Animal Care. This study conformed to the standards of the ARVO Statement for the Use of Animals in Ophthalmic and Vision Research. All experiments involving animals were approved by the Science and Technology Ethics Committee of The Second Affiliated Hospital of Chongqing Medical University (2021354). Forty male New Zealand white rabbits (2.0 to 2.5 kg, 14 to 16 weeks of age) were obtained from the Animal Experiment Center of Chongqing Medical University (Chongqing, China). Rabbits with ocular pathologies or diseases were excluded from the study. The left eyes of 36 rabbits underwent refractive corneal surgery (transepithelial photorefractive keratectomy [TransPRK]) using an excimer laser corneal refractor (STAR S4 IR, AMO Manufacturing USA, LLC). The degree and range of laser ablation were −9.00 D for the sphere and an optical zone of 6.00 mm. The rabbits were anesthetized by auricular vein injection of pentobarbital sodium (3%, 1 ml/kg). In this study, the rabbits were randomly and evenly divided into 6 groups: normal, control (PBS), TiO_2_, Tet, fluorometholone eye drops (FML), and Tet@TiO_2_.

### Biosafety of FML, Tet, and Tet@TiO_2_

Human corneal stromal fibroblasts (HCSFs) were purchased from Shanghai Sixin Biotechnology Co., Ltd. in China, and their acquisition and purchase were approved and complied with ethical drug regulations on the basis of normal procedures. The HCSFs were seeded into 96-well plates at a density of 5.0 × 10^4^ cells per well and cultured for 24 h. Next, the cells were divided into 9 groups, with each group consisting of 5 wells. FML, Tet, and Tet@TiO_2_ were added to the corresponding experimental wells at final concentrations of 0, 2, 4, 6, 8, 10, 20, 30, and 40 μg/ml, while fresh culture medium was added to the control group. After incubation for 24 h, cell viability was quantified by a Cell Counting Kit-8 (CCK-8) assay. For the live/dead cell staining experiment, 5 × 10^5^ HCSFs were cultured in a laser confocal cell culture dish, and the appropriate concentrations of FML, Tet, and Tet@TiO_2_ were added after 24 h of incubation. After 24 h of coincubation, all of the samples were observed by confocal laser scanning microscopy (CLSM; Nikon A1, Japan) after staining with M5 HiPer calcein AM/propidium iodide (Beijing, China). In addition, flow cytometry (FCM; FACS Vantage, Becton Dickinson, USA) was used to determine the cell survival rates after 24 h of drug treatment.

### In vivo biosafety evaluation

New Zealand white rabbits were randomly divided into 5 groups, normal control, TiO_2_, FML, Tet, and Tet@TiO_2_, and all of the rabbits were subjected to simulated ophthalmic clinical medication application. One drop (15 μl) of TiO_2_, FML, Tet, or Tet@TiO_2_ at the same concentration (1 mg/ml) was added to the right eye of each rabbit 3 times a day (0800, 1600, and 2400), whereas the rabbits in the normal control group were not treated. A slit lamp was subsequently used to observe corneal fluorescence staining and the conjunctiva, anterior chamber, lens, and other structures. The intraocular pressure of each rabbit was measured on the 15th and 30th days of simulated clinical medication application using a rebound tonometer. After 30 d of treatment, blood was collected from the ear vein of each rabbit using a syringe and placed into an EDTA-K2 anticoagulant tube. The blood samples were either subjected to routine blood analysis using a fully automated blood analyzer or placed in a centrifuge tube and allowed to stand at room temperature for 2 h. The latter samples were subsequently centrifuged for 10 min in a low-temperature centrifuge (4 °C, 3,000 rpm), after which biochemical analysis was performed. The white blood cell (WBC), lymphocyte (LYM), and platelet (PLT) counts; hemoglobin (HGB), aspartate transaminase (AST), alanine transaminase (ALT), blood urea nitrogen (BUN), fasting blood glucose, triglyceride, total cholesterol, high-density lipoprotein cholesterol, low-density lipoprotein cholesterol, and creatinine (Crea) levels; and estimated glomerular filtration rate (eGFR) were determined, among other parameters. Then, pentobarbital sodium (3%, 1 ml/kg) was injected into the ear vein of each rabbit for general anesthesia, after which high concentrations of CO_2_ were administered to suffocate the animals for sacrifice. The eyeballs were removed, and the heart, liver, spleen, lungs, and kidneys were collected for fixation, embedding, and hematoxylin and eosin (H&E) staining.

#### Assessment of the bactericidal effects of Tet@TiO_2_

The bactericidal efficacy of Tet@TiO_2_ against methicillin-resistant *Staphylococcus aureus* (MRSA), *Escherichia coli*, and methicillin-resistant *Staphylococcus epidermidis* (MRSE) was evaluated by qualitative detection and plate counting methods. First, 200 μl of bacterial suspension was incubated for 24 h. Then, tobramycin dexamethasone eye drops (TDED; Alcon, Belgium), levofloxacin eye drops (LED; Beijing, China), FML, or Tet@TiO_2_ was added to the cells in the experimental groups to evaluate their bactericidal properties. Next, the number of bacteria remaining at different time points after the addition of LED, TDED, Tet@TiO_2_, and chloramphenicol eye drops (CED; Handan, China) was determined by plate counting. Finally, the bactericidal properties of the drugs were quantitatively determined using the following equation:

$$\begin{array}{c} \text{Bactericidal rate}\;\left(S\right)=M/N_{0}\times 100\%, \end{array}$$ (4)

where *N* is the number of bacterial colonies at the corresponding time point after drug administration, *N*_0_ is the number of bacterial colonies present before drug administration (0 h), and *M* represents the number of dead bacteria after drug administration (*M* = *N*_0_ − *N*).

#### Cellular uptake of Tet@TiO_2_

The in vitro uptake of Tet@TiO_2_ by HCSFs was evaluated via CLSM and FCM. Inflammatory HCSFs were treated with TGF-β_2_ (4 ng/ml) for 24 h. Additionally, DiI (*λ* excitation, 549 nm; *λ* emission, 565 nm)-labeled Tet@TiO_2_ (150 μg/ml) was added to normal and inflammatory HCSFs. After incubation for 0.5, 1, and 2 h, the cells were washed to remove noninternalized particles and fixed with 4% formaldehyde, and the nuclei were stained with 4′,6-diamidino-2-phenylindole (DAPI; *λ* excitation, 364 nm; *λ* emission, 454 nm). The changes in cell morphology after drug uptake were subsequently observed by CLSM (Nikon, Tokyo, Japan). In addition, the cellular uptake of DiI-labeled nanoparticles was quantified by FCM after different time intervals.

#### Infiltration and distribution of Tet@TiO_2_ in the rabbit corneal stroma

First, DiI-labeled Tet@TiO_2_ (1 mg/ml) was added to the ocular surface dropwise, and the rabbits were placed in a dark environment. After 0.5, 1, and 2 h, the rabbit corneas were removed and fixed with 10% neutral buffered formalin. The eyeballs were then frozen on dry ice and stored at −80 °C. The frozen eyeballs were subsequently washed twice with PBS to remove the frozen media and then stained with DAPI for 30 min. The frozen corneas were cut into 10-μm sections with a cryostat microtome (CM1860, Leica, Germany), and the cryosections were observed and photographed under a fluorescence microscope (Eclipse Ti-S, Nikon, Japan).

#### Effects on tissue morphology

The rabbits were divided into the following groups: normal, control (PBS), TiO_2_, FML, Tet, and Tet@TiO_2_. Recovery of the corneal epithelium was continuously observed for 5 d after surgery after the addition of 2 μl of 1% fluorescein sodium. After the corneal epithelium had healed, the appropriate drug was added to each experimental group, and the development of and changes in corneal haze were recorded and photographed after 2 weeks of treatment. Then, the corneas were fixed with paraformaldehyde (4% in PBS) for 1 h, embedded in paraffin, and cut into sections. The target area of the corneal tissue was observed with an Eclipse ci-l polarized light microscope, and the Image-Pro Plus 6.0 analysis software was used to measure the areas of type I and type III collagen fibers in each image, using pixels as the standard unit. Furthermore, TEM was applied to observe the macroscopic and microstructural changes in the cornea before and after treatment. The development of corneal haze was graded as follows: (a) level 0, the cornea is completely transparent; (b) level 0.5, the cornea is slightly turbid and can be seen only in indirect light with a slit lamp; (c) level 1, the cornea shows low-density turbidity that is visible by direct illumination or diffuse illumination with a slit lamp; (d) level 2, vision is affected by slight corneal turbidity that can be seen with direct focusing using a slit lamp; (e) level 3, moderate corneal opacity covering part of the iris; and (f) level 4, the cornea is severely turbid and blocks the tissue structures of the eye.

#### Comparative analysis of the anti-inflammatory effects of FML, Tet, and Tet@TiO_2_ and exploration of the mechanism of Tet@TiO_2_ in vitro

Immunohistochemistry and Western blotting (WB) were used to compare the changes in the expression of the inflammatory cytokines α-SMA and CTGF and type III collagen in vitro. Immunohistochemical staining of α-SMA, CTGF, and type III collagen in the corneal tissues was performed. Proteins were subsequently extracted from the corneal tissues of the rabbits in each group, and WB was conducted to evaluate the expression of the above 3 proteins. Then, immunohistochemistry and WB were used to measure the changes in the expression of PI3K, AKT, Bcl-2, and Bax and the Bax/Bcl-2 ratio in each group. Primary antibodies against α-SMA, CTGF, type III collagen, PI3K, AKT, Bcl-2, and Bax were purchased from Wuhan Doctoral Biotechnology Co., Ltd. (China) and used for immunohistochemistry and WB after being diluted 1:1,000.

### RT-qPCR analysis of the in vivo effects of Tet@TiO_2_ on the PI3K–AKT–Bax/Bcl-2 inflammatory signaling pathway

The RNA expression of the key inflammatory factors PI3K, AKT, Bax, and BCL-2 in the corneal tissues from each group were measured via real-time quantitative polymerase chain reaction (RT-qPCR) (Table).

**Table.** The PI3K, AKT, Bax, and Bcl-2 primer sequences used for the in vivo RT-qPCR experiments

Primer	F or R	Primer sequence	Product length (bp)
GAPDH	F	5′-TTCCAGTATGATTCCACCCACG-3′	232
	R	3′-GGGCTGAGATGATGACCCTTTT-5′	
PI3K	F	5′-CTTCCTCGTGCTGCTCGACTT-3′	214
	R	3′-TTGGATTTGACCCAGTAACACC-5′	
AKT	F	5′-CCTGAGGTGCTGGAGGACAA-3′	117
	R	3′-CTTCTCGTGGTCCTGGTTGTAG-5′	
Bax	F	5′-GCTTCAGGGTTTCATCCAGTATC-3′	201
	R	3′-ATCCTCTGCAGCTCCATGTTACT-5′	
Bcl-2	F	5′-GCATTGTGGCCTTCTTTGAGTTC-3′	125
	R	3′-CTTCAGAGACAGCCAGGAGAAAT-5′	

PI3K, phosphatidylinositol 3-kinase; AKT, protein kinase B; Bax, Bcl-2-associated X protein; Bcl-2, B-cell lymphoma-2; RT-qPCR, real-time quantitative polymerase chain reaction; F, forward; R, reverse; GAPDH, glyceraldehyde-3-phosphate dehydrogenase

### Statistical analysis

Statistical analysis was performed using GraphPad Prism 8.1 (California, USA). Quantitative data are reported as mean ± standard deviation (SD). A 2-tailed Student *t* test was used to compare 2 groups, while one-way analysis of variance (ANOVA) followed by Tukey’s multiple comparisons test was used for multigroup comparisons. A value of *P* < 0.05 was considered to indicate statistical significance.

## Results

### Fabrication and characterization of Tet@TiO_2_

**Fig. 1.**
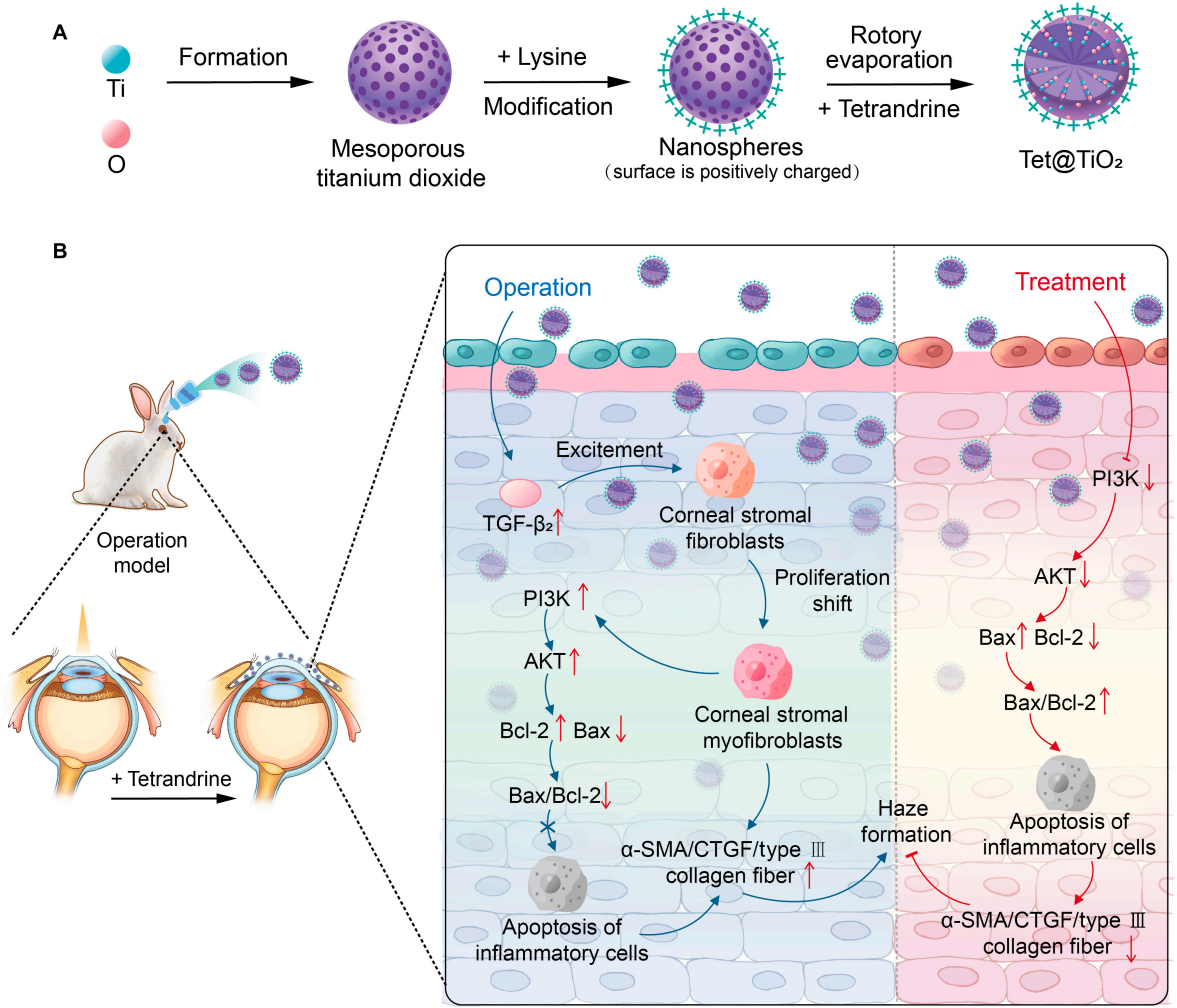
(A) Composite diagram of Tet@TiO_2_. (B) Schematic of the Tet@TiO_2_ treatment process. Tet, tetrandrine; TGF-β_2_, transforming growth factor-β_2_; α-SMA, α-smooth muscle actin; CTGF, connective tissue growth factor.

**Fig. 2.**
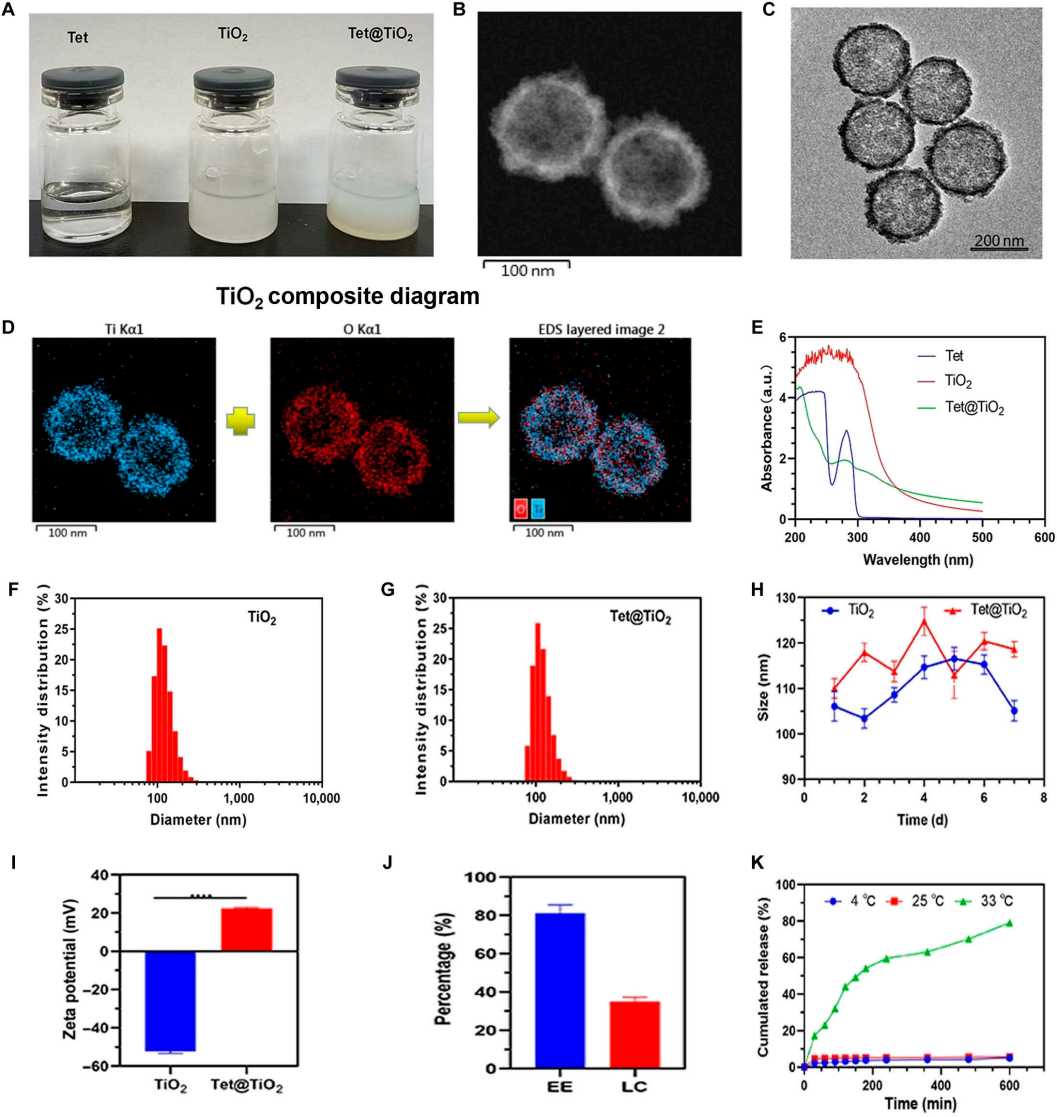
(A) Appearances of Tet, TiO_2_, and Tet@TiO_2_. (B and C) Transmission electron microscopy (TEM) images of TiO_2_ and Tet@TiO_2_ (200 μg/ml). (D) Composite diagram of TiO_2_. (E) Ultraviolet–visible–near-infrared (UV–vis–NIR) absorbance spectra of free Tet, TiO_2_, and Tet@TiO_2_ (50 μg/ml). (F and G) Size distributions of TiO_2_ and Tet@TiO_2_ (50 μg/ml). (H) TiO_2_ and Tet@TiO_2_ size distributions after prolonged storage in phosphate-buffered saline (PBS). (I) Zeta potentials of TiO_2_ and Tet@TiO_2_ (50 μg/ml). (J) Encapsulation efficiency (EE) and drug loading capacity (LC) of Tet@TiO_2_. (K) Cumulative in vitro release of Tet from Tet@TiO_2_ in PBS at 4, 25, and 33 °C. Linear regression, *t* tests, and means and SDs were used for analysis (*P* < 0.05). EDS, energy-dispersive x-ray spectroscopy.

A schematic of drug synthesis is provided in Fig. 1A. As shown in Fig. 2A, both Tet@TiO_2_ and TiO_2_ appeared milky white in PBS, whereas the Tet solution was transparent. In addition, TEM revealed that TiO_2_ had a circular structure with mesopores on the surface and that the surface of Tet@TiO_2_ was rough due to Tet loading (Fig. 2B and C). As shown in Fig. 2D, TiO_2_ was synthesized from Ti (blue) and O (red). Moreover, the UV–visible–near-infrared spectra of Tet and Tet@TiO_2_ presented a characteristic absorption peak at 282 nm, whereas the spectrum of TiO_2_ did not (Fig. 2E). In addition, dynamic light scattering analysis indicated that the average diameters of TiO_2_ and Tet@TiO_2_ were 108.27 ± 11.72 and 120.81 ± 9.74 nm, respectively, and both had favorable polydispersity indices of 0.183 and 0.157, respectively (Fig. 2F and G). Moreover, the average particle sizes of TiO_2_ on each day during 1 week of storage were 106.03 ± 3.25, 103.41 ± 2.17, 108.63 ± 1.63, 114.67 ± 2.53, 116.43 ± 2.51, 115.23 ± 2.11, and 105.13 ± 2.25 nm, whereas those of Tet@TiO_2_ were 110.07 ± 2.15, 117.97 ± 2.06, 113.76 ± 2.26, 124.77 ± 3.11, 122.1 ± 5.21, 120.41 ± 1.95, and 118.63 ± 1.71 nm. These changes in the sizes of TiO_2_ and Tet@TiO_2_ over 7 d revealed that both materials were stable at room temperature (Fig. 2H). The average zeta potentials of TiO_2_ and Tet@TiO_2_ were −52.23 ± 1.47 and 22.47 ± 0.62 mV, respectively. Additionally, the surface of TiO_2_ was positively charged after lysine modification [36,37], which allows Tet@TiO_2_ to better adapt to the negatively charge on the eye surface (Fig. 2I). The EE and LC of Tet@TiO_2_ were calculated to be 81.18% ± 4.41% and 35.02% ± 2.28%, respectively (Fig. 2J), on the basis of the standard curve constructed from the Tet UV absorption data (*Y* = 11,786.8*X* − 540.966; *R*^2^ = 0.9986). Moreover, the cumulative release of Tet from Tet@TiO_2_ over 600 min was analyzed via high-performance liquid chromatography, and the maximum release rates were 5.12% ± 0.23%, 5.62% ± 0.17%, and 79.15% ± 0.87% at 4, 25, and 33 °C, respectively (Fig. 2K). These results indicated the good release performance of Tet from Tet@TiO_2_ at corneal temperature and that the formulation was stable during storage at 4 °C and at room temperature.

### Viability of HCSFs treated with FML, Tet, and Tet@TiO_2_

**Fig. 3.**
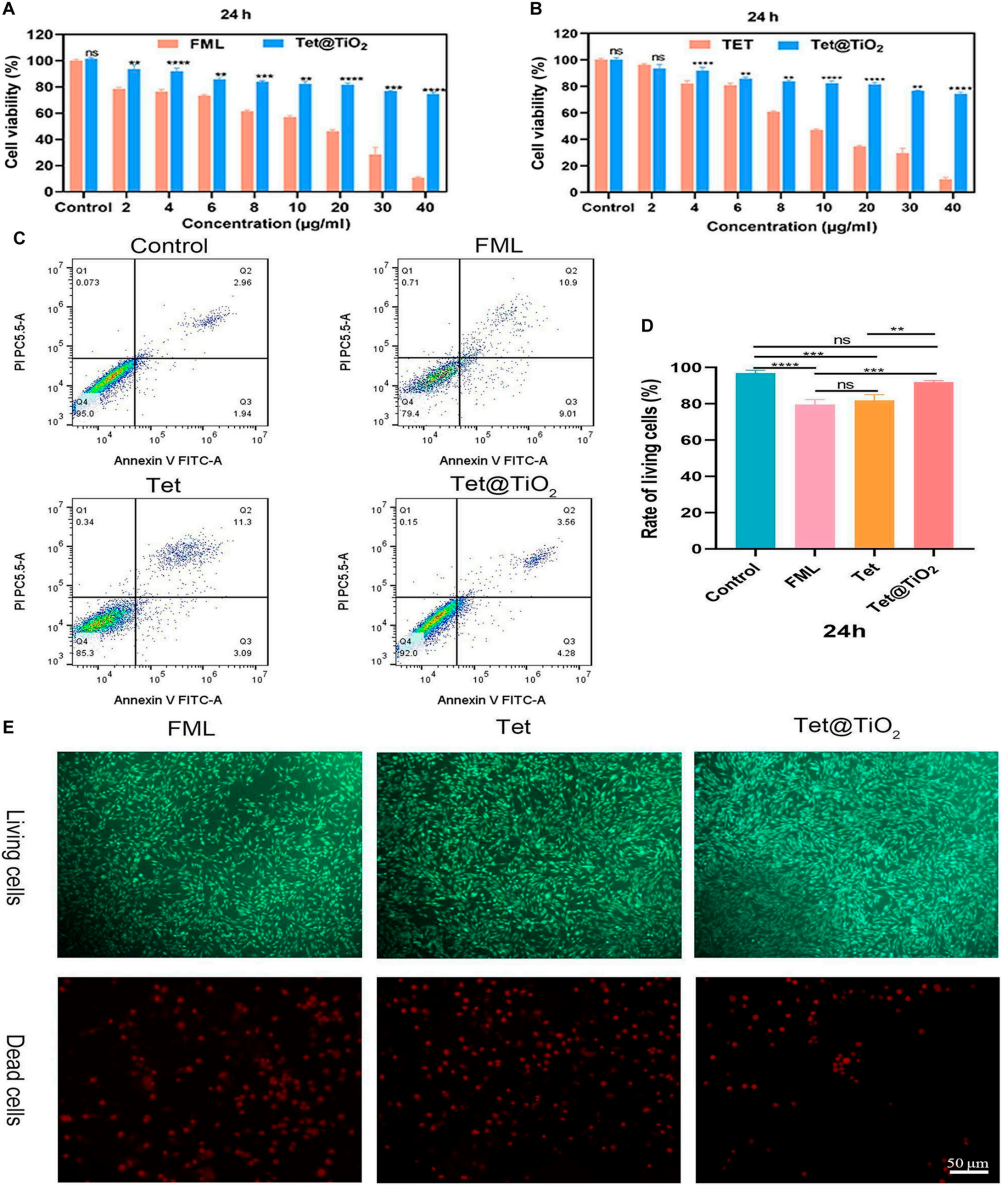
(A and B) Viability of human corneal stromal fibroblasts (HCSFs) after treatment with various concentrations of Tet, fluorometholone eye drops (FML), or Tet@TiO_2_ for 24 h. (C and D) Rates of living HCSFs after 24 h of incubation with 4 μg/ml Tet, FML, and Tet@TiO_2_. (E) Live/dead staining images of HCSFs after 24 h of incubation with 4 μg/ml Tet, FML, and Tet@TiO_2_. Ordinary one-way ANOVA; **P < 0.01; ***P < 0.001; ****P < 0.0001; ns, not significant.

CCK-8 assays, FCM, and M5 HiPer calcein AM/propidium iodide staining were employed to determine if FML, Tet, and Tet@TiO_2_ were toxic to HCSFs at concentrations ranging from 0 to 40 μg/ml, wherein for Tet@TiO_2_, the concentration of encapsulated Tet was used to determine the Tet@TiO_2_ concentration. As shown in Fig. 3A and B, cell viability decreased as the concentrations of FML and Tet increased in a dose-dependent manner. However, cell viability decreased significantly more slowly after treatment with Tet@TiO_2_ than after treatment with FML or Tet. After incubation for 24 h with the maximum drug concentration (40 μg/ml), 78.23% ± 3.49% of the cells treated with Tet@TiO_2_ were viable, whereas 12.27% ± 2.81% and 9.86% ± 1.92% of the cells were viable after treatment with FML and Tet, respectively. Clearly, the influence of Tet@TiO_2_ on cell viability remained low even when it was administered at high concentrations. Then, HCSFs were seeded into a 6-well plate, and after they had matured, 4 μg/ml FML, Tet, or Tet@TiO_2_ was added for 24 h of incubation, after which FCM was used to detect the number of living cells. As shown in Fig. 3C and D, the percentages of living cells in the control, FML, Tet, and Tet@TiO_2_ groups were 95.12% ± 0.87%, 80.09% ± 1.37%, 85.61% ± 1.62%, and 92.16% ± 0.78%, respectively. The FCM data clearly revealed that, compared with FML and Tet, Tet@TiO_2_ was much less toxic to HCSFs. In addition, after performing live/dead staining, wherein the green fluorescent cells were alive and the red fluorescent cells were dead, fewer dead cells were observed in the Tet@TiO_2_ group than in the FML and Tet groups, indicating that at the same concentration, Tet@TiO_2_ was much safer than FML and Tet (Fig. 3E). On the basis of these CCK-8, FCM, and live/dead staining data, we confirmed the better safety of Tet@TiO_2_.

### In vivo biosafety of Tet@TiO_2_

**Fig. 4.**
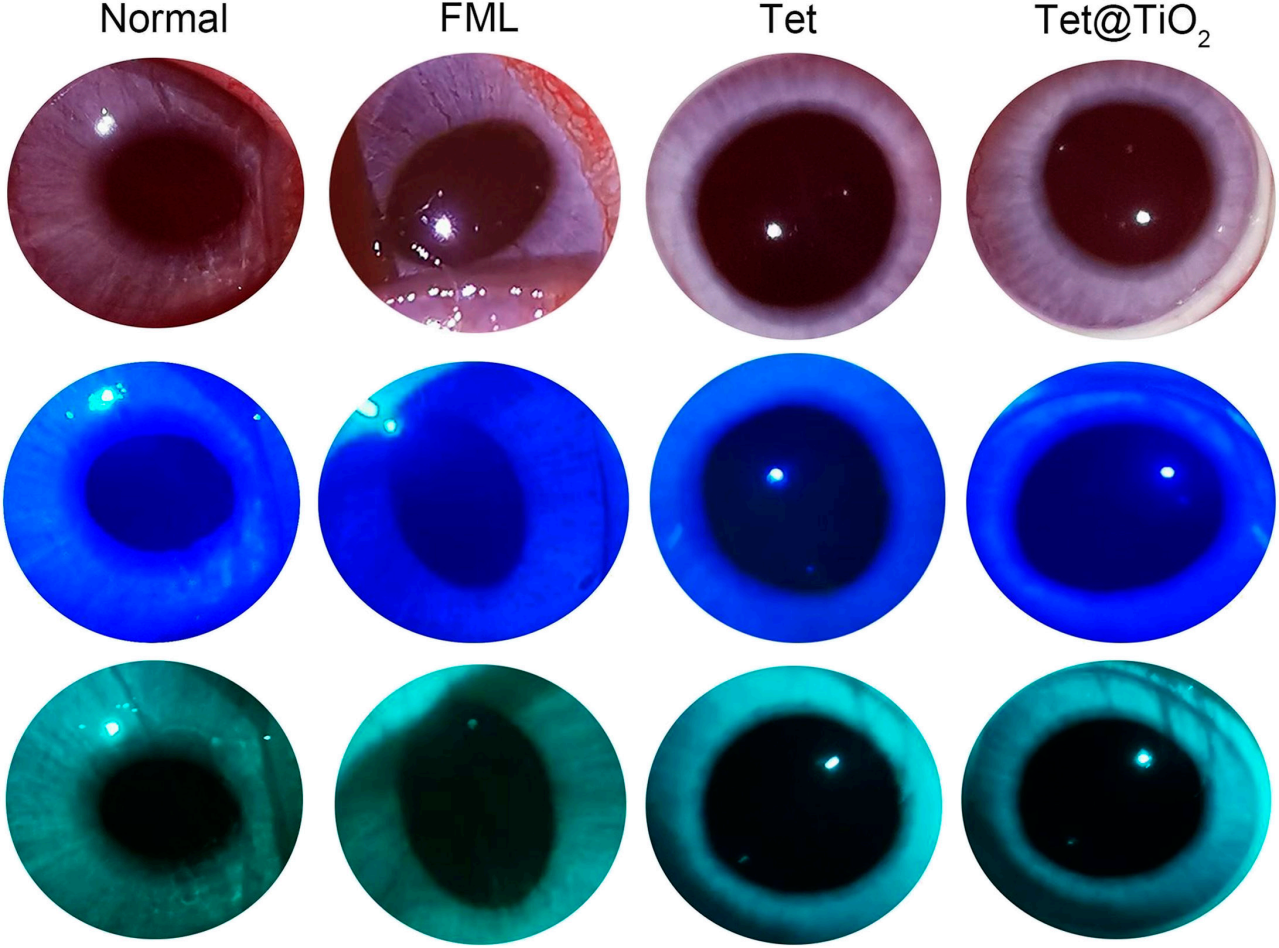
Slit lamp imaging and corneal fluorescence staining revealed no abnormalities in the eyes of the New Zealand white rabbits in the normal, FML, Tet and Tet@TiO_2_ groups after 30 d of treatment.

The rabbits were divided into normal, FML, Tet, and Tet@TiO_2_ groups, and their right eyes were treated with 15 μl of the appropriate material 3 times a day for 30 d. Then, a slit lamp was used to observe conjunctival congestion, secretions, corneal transparency, corneal neovascularization, and corneal fluorescence staining. There were no significant differences in any of these parameters among the groups (Fig. 4).

**Fig. 5.**
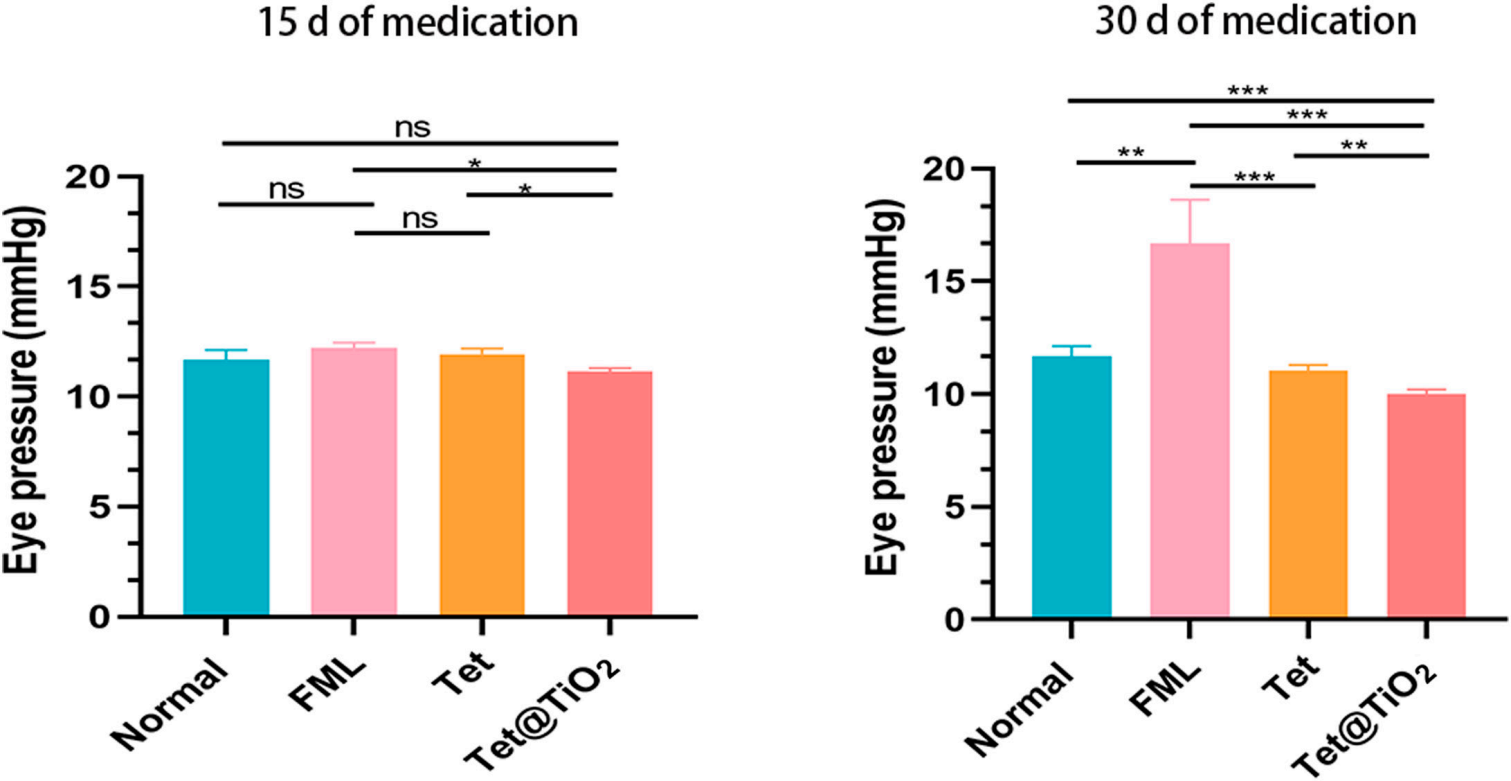
Changes in the eye pressure (mmHg) of the New Zealand white rabbits in the normal, FML, Tet, and Tet@TiO_2_ groups after 15 and 30 d of treatment.

Additionally, a rebound tonometer was used to measure the intraocular pressure in the eyes of the New Zealand white rabbits in the normal, FML, Tet, and Tet@TiO_2_ groups. The intraocular pressure in the FML group after 15 d and in the Tet group after 30 d was not significantly different (*P* > 0.05) from that of the normal group (11.67 ± 0.45 mmHg). Moreover, although there was no significant difference in the intraocular pressure between the FML group (12.2 ± 0.26 mmHg) and the Tet group (11.93 ± 0.25 mmHg) after 15 d of treatment, the intraocular pressure in the Tet@TiO_2_ group (11.13 ± 0.15 mmHg) was significantly lower (*P* < 0.05). Further analysis of the changes in intraocular pressure in each group after 30 d of treatment revealed that, compared with that of the normal group (11.67 ± 0.45 mmHg), the intraocular pressure in the FML group (16.67 ± 1.96 mmHg) was significantly greater, whereas those in the Tet (11.03 ± 0.25 mmHg) and Tet@TiO_2_ groups (10.12 ± 0.2 mmHg) continued to decrease. Furthermore, the decrease in intraocular pressure in the Tet@TiO_2_ group was significantly different, reaching 1.45 ± 0.17 mmHg. Compared with long-term FML use, which can easily lead to increased intraocular pressure, Tet administration did not increase intraocular pressure; in fact, Tet reduced the intraocular pressure. After being encapsulated in the nanoplatform, Tet release was sustained, which further enhanced the ability of Tet to reduce intraocular pressure (Fig. 5).

**Fig. 6.**
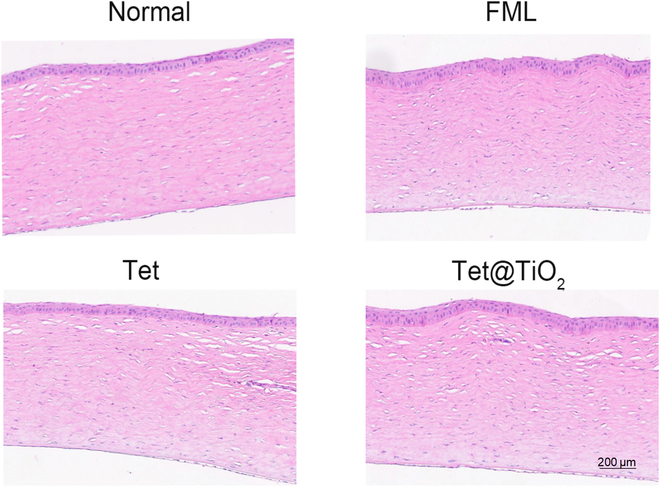
Hematoxylin and eosin (H&E) staining images of corneal tissue from the normal, FML, Tet, and Tet@TiO_2_ groups after 30 d of treatment revealed no abnormalities.

After treating the ocular surfaces of the New Zealand white rabbits in the normal, FML, Tet, and Tet@TiO_2_ groups for 30 d, H&E staining was performed on the corneas for pathological analysis. The results revealed that in each group, the structure of each layer of corneal tissue was intact and tightly arranged, there was no notable corneal edema or changes in cell morphology, and no obvious abnormal events, such as inflammatory cell infiltration, were observed (Fig. 6).

**Fig. 7.**
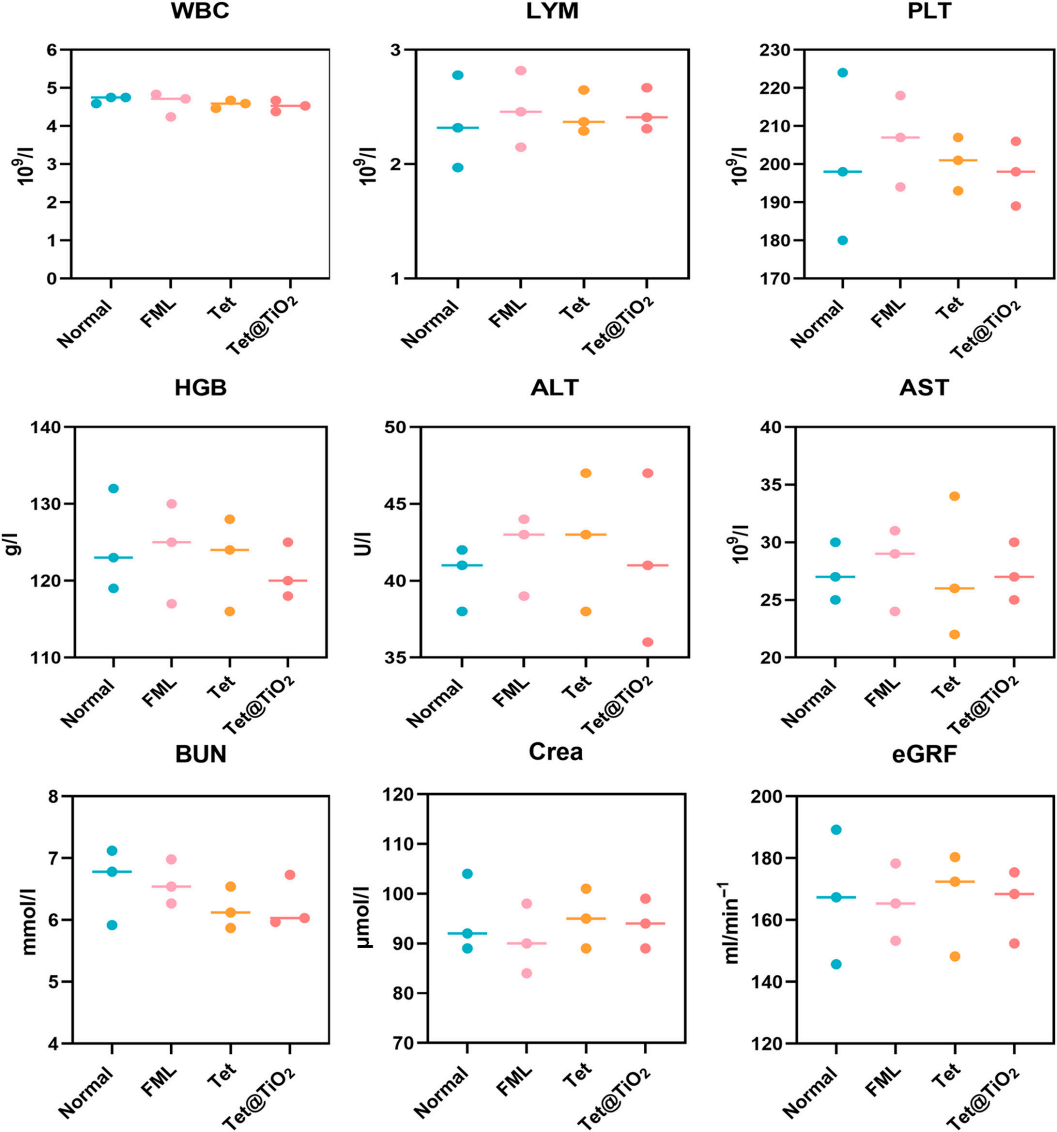
Routine blood, liver, and kidney function indices after 30 d of treatment revealed no abnormalities among the normal, FML, Tet, and Tet@TiO_2_ groups (*n* = 5/group). WBC, white blood cell; LYM, lymphocyte; PLT, platelet; HGB, hemoglobin; ALT, alanine transaminase; AST, aspartate transaminase; BUN, blood urea nitrogen; Crea, creatinine; eGFR, estimated glomerular filtration rate.

After treating the ocular surfaces of the New Zealand white rabbits for 30 d, venous blood was collected from the fasted rabbits for routine blood and liver and kidney function tests. There were no significant differences in measured indicators, such as the WBC, LYM, or PLT counts; the HGB, ALT, AST, Crea, or BUN levels; or the eGFR among the normal, the FML, Tet, and Tet@TiO_2_ groups. Thus, these data indicated that the above treatments were safe to administer to New Zealand white rabbits (Fig. 7).

**Fig. 8.**
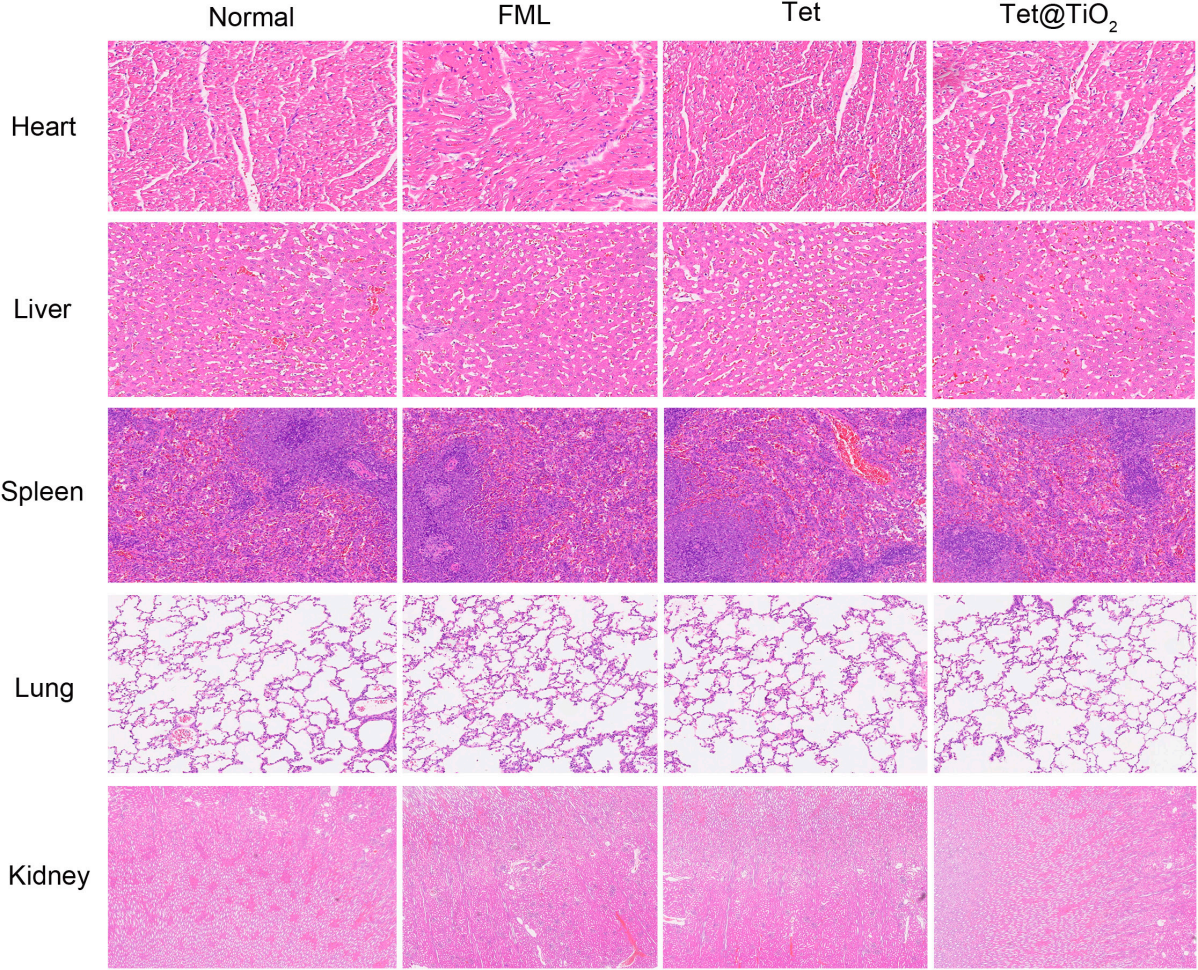
H&E staining images of the major organs after 30 d of treatment revealed no abnormalities among the normal, FML, Tet, and Tet@TiO_2_ groups.

After the drugs had been administered for 30 d after TransPRK, pentobarbital sodium (3%, 1 ml/kg) was administered to the rabbits in the normal, FML, Tet, and Tet@TiO_2_ groups; anesthesia was administered via the ear vein; and the main organs (heart, liver, spleen, lungs, and kidneys) were removed. Compared with the normal group, the FML, Tet, and Tet@TiO_2_ groups presented no significant pathological changes in the main organs, indicating that the above treatments did not have any adverse effects on the New Zealand white rabbit main organ tissue structures (Fig. 8).

### Tet@TiO_2_ accumulation and targeting in a corneal haze model in vivo and inflammatory HCSFs in vitro

**Fig. 9.**
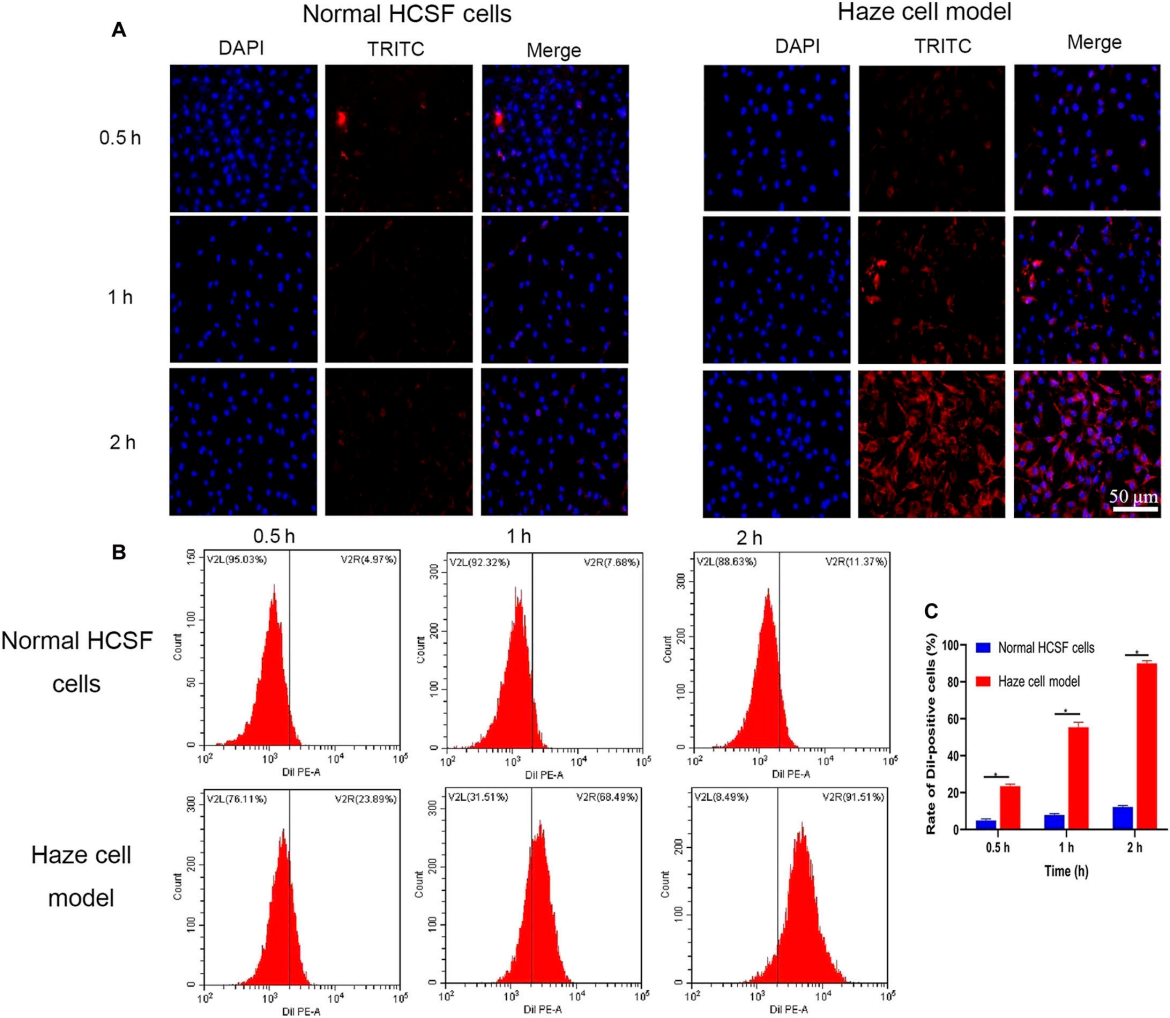
(A) Uptake of DiI-labeled Tet@TiO_2_ (150 μg/ml) by normal and inflamed HCSFs observed by confocal laser scanning microscopy (CLS)M after different durations. (B) Flow cytometry detection of the uptake of DiI-labeled Tet@TiO_2_ (150 μg/ml) after different durations. (C) Quantitative analysis via flow cytometry. Multiple *t* tests. DAPI, 4′,6-diamidino-2-phenylindole.

CLSM and FCM were employed to precisely measure the accumulation and targeting effects of Tet@TiO_2_ in vivo and in vitro. As shown in Fig. 9A, DiI-labeled Tet@TiO_2_ was present mainly in inflamed HCSFs and to a lesser extent in normal HCSFs, with the highest DiI signal observed after 2 h. Furthermore, FCM analysis revealed that the uptake efficiency of DiI-labeled Tet@TiO_2_ by normal cells was 4.97%, 7.68%, and 11.37%, whereas that by inflamed cells was 23.89%, 68.49%, and 91.51% at 0.5, 1, and 2 h, respectively (Fig. 9B and C). These results showed that Tet@TiO_2_ phagocytosis by HCSFs increased significantly after TGF-β_2_ stimulation. In addition, DiI-labeled Tet@TiO_2_ gradually penetrated from the corneal epithelium to the corneal stroma in the haze model after TransPRK, as observed in the frozen corneal sections, and the DiI fluorescence signal was distributed diffusely in the corneal stroma layer; moreover, the intensity of the fluorescence signal was strongest after 2 h. However, there was only weak DiI fluorescence in the corneal stroma of normal eyes. Therefore, less Tet@TiO_2_ entered the corneal stroma layer; however, Tet@TiO_2_ was distributed diffusely in the corneal haze formed in the surgical area after TransPRK, indicating that Tet@TiO_2_ effectively targeted eyes with corneal haze (Fig. S1).

### Histopathological manifestations after Tet@TiO_2_ hindered haze development in rabbit corneas after TransPRK

As shown in the corneal staining images in Fig. S2a, the corneal epithelium returned to normal on the fifth day after the operation, and corneal haze began to appear on the seventh day after TransPRK. After corneal haze reached the level 2 classification, the right eyes of the 36 rabbits were divided into the control (normal saline), TiO_2_, FML, Tet, and Tet@TiO_2_ groups, with 6 rabbit eyes in each group. Additionally, 6 rabbit eyes that had not undergone TransPRK were included in the normal group. One week after the operation, when corneal haze was classified as level 2, the corneas in each group were treated with the appropriate drug. As shown in Fig. S2b, the corneas in the control, TiO_2_, FML, Tet, and Tet@TiO_2_ groups showed different extents of recovery at 2 weeks after the operation: the corneas treated with Tet@TiO_2_ were classified as level 0, whereas the corneas in the control, TiO_2_, FML, and Tet groups were level 2, level 2, level 1, and level 0.5, respectively. After the corneas were stained with H&E, the corneal epithelium and stromal layer in the control and TiO_2_ groups presented notable edema, and there were obvious cracks between them. Furthermore, the basement membrane was discontinuous, the basal cells were arranged irregularly, the collagen fibers were disordered, and the epithelial basal cells exhibited vacuolar degeneration; these structural changes easily led to the development of corneal haze. Compared with the FML and Tet groups, the Tet@TiO_2_ group showed better results, as follows: (a) Edema in the corneal epithelium and stroma was reduced, there were no cracks between the epithelial layer and the parenchyma layer, the basement membrane was continuous and uniform in thickness, and the cornea nearly returned to a normal shape. (b) Basal cell vacuolar degeneration disappeared when the corneal thickness and number of corneal stromal cells returned to essentially normal. (c) The collagen fibers were close together and arranged regularly (Fig. S2c). Moreover, the type I collagen fibers appeared yellowish red, whereas the type III collagen fibers appeared green under a polarized light microscope. The areas with positive staining for type I and III collagen clearly showed the substantial expression of these markers after surgery in the control and TiO_2_ groups, with type III collagen being the most obvious (Fig. S3a). After administration, the areas positively stained for type I and III collagen fibers in the Tet@TiO_2_ group were smaller than those in the FML and Tet groups, and there was no significant difference between the latter groups (*P* = 0.271). These results showed that compared with FML and Tet, Tet@TiO_2_ more effectively reduced the expression of type I and III collagen after refractive corneal surgery (Fig. S3b).

Further TEM observations 3 weeks after TransPRK revealed the following in the control group: (a) Many myofibroblasts were distributed in the stromal layer of the surgical area. (b) The cell volume and nuclei were large, while the extracellular collagen fibers were uneven and disorderly arranged. (c) The cells were rich in rough endoplasmic reticula, mitochondria, Golgi apparatuses, and other organelles, and the basement membrane was thin and discontinuous. The above results indicated that vigorous metabolism and active proliferation occurred in the control group, which easily led to the development of corneal haze. Compared with those in the FML and Tet groups, the corneas in the Tet@TiO_2_ group were nearly normal in shape after 2 weeks of treatment, as evidenced by the following: (a) The extracellular collagen fibers were more uniform in thickness, and their arrangement was more regular. (b) There were few myofibroblasts in the anterior stromal layer. (c) The cell volume and nuclei in the edematous areas were significantly smaller. (d) The rough endoplasmic reticula, mitochondria, and Golgi apparatuses returned to near normal. The cells in the Tet@TiO_2_ group clearly displayed weak metabolic and proliferative activities, so corneal haze did not develop. These results demonstrated that the cornea ultrastructure in the Tet@TiO_2_ group recovered quickly after the operation compared with those in the FML and Tet groups after the same treatment duration (Fig. S3c). In summary, the above results indicated that Tet@TiO_2_ can effectively treat corneal haze and that its effect is better than those of FML or Tet after 2 weeks of treatment.

### Assessment of the anti-inflammatory effects of Tet@TiO_2_ in rabbit eyes after TransPRK

In this study, the expression of α-SMA, CTGF, and type III collagen in all groups was measured directly via immunohistochemistry and WB at 3 weeks after TransPRK. We graded the intensity of expression immunohistochemically as follows: level 0, no positive staining; level 1, weakly positive, light yellow; level 2, positive, brown-yellow; and level 3, strongly positive, brown. As shown in Fig. S4a, the α-SMA, CTGF, and type III collagen expression in the control and TiO_2_ groups was classified as level 3, whereas their expression was classified as level 2, level 2, and level 1 in the FML, Tet, and Tet@TiO_2_ groups, respectively. The expression intensities of the inflammation-related factors in the Tet@TiO_2_ group were clearly similar to those in the normal group. Furthermore, we calculated and statistically analyzed the H scores of the inflammation-related antibodies as follows: H score = ∑(*pi* × *i*) = (percentage with weak intensity × 1) + (percentage with moderate intensity × 2) + (percentage with strong intensity × 3). For example, compared with the H score of α-SMA in the normal group (10.49 ± 2.01), those in the control group (42.31 ± 2.74) and TiO_2_ group (38.81 ± 3.99) were significantly greater, but the α-SMA H score decreased after FML (25.76 ± 2.54), Tet (20.41 ± 1.84), and Tet@TiO_2_ (15.28 ± 1.84) treatment, among which Tet@TiO_2_ showed the most obvious decrease (Fig. S4c). Surprisingly, the changes in the H scores of type III collagen and CTGF showed the same trend (Fig. S4b and d).

Furthermore, after protein was extracted from the corneal tissue of each group at 3 weeks after TransPRK for WB, the expression of α-SMA, CTGF, and type III collagen was significantly lower in the FML, Tet, and Tet@TiO_2_ groups than in the control and TiO_2_ groups. In addition, the levels of inflammatory cytokines released in the Tet@TiO_2_ group were lower than those in the FML and Tet groups (Fig. S4e to h). In summary, we believe that Tet@TiO_2_ effectively reduces the expression of the inflammatory factors α-SMA, CTGF, and type III collagen in rabbit eyes after TransPRK, thus inhibiting the development of corneal haze. When the immunohistochemistry and WB results were considered together, the therapeutic effect of Tet@TiO_2_ was better than those of FML and Tet.

### Exploration of the mechanism by which Tet@TiO_2_ hinders haze development in vivo after TransPRK

The mechanism of action of Tet@TiO_2_ was evaluated by immunohistochemistry and WB, and a diagram of its mechanism of action is shown in Fig. 1B. First, we conducted immunohistochemical analysis on corneal tissue from each group at 3 weeks after TransPRK. As shown in Fig. S5a, the intensities of PI3K, AKT, and Bcl-2 expression in the control and TiO_2_ groups were classified as level 3, whereas these factors were classified as level 2, level 2, and level 1 in the FML, Tet, and Tet@TiO_2_ groups, respectively. Moreover, the intensity of Bax expression in the control and TiO_2_ groups was level 1, whereas it was level 2, level 2, and level 3 in the FML, Tet, and Tet@TiO_2_ groups, respectively. Furthermore, we analyzed the PI3K, AKT, Bcl-2, and Bax H scores. The results revealed that the H scores of PI3K were significantly greater in the control group (41.21 ± 1.12) and TiO_2_ group (40.02 ± 3.35) than in the normal group at 3 weeks after TransPRK, whereas the H scores decreased after FML, Tet, and Tet@TiO_2_ treatment, with the most obvious decrease in the Tet@TiO_2_ group (8.49 ± 0.69) (Fig. S5b). Moreover, AKT and Bcl-2 were detected in the normal, control, TiO_2_, FML, Tet, and Tet@TiO_2_ groups, and the H scores in these groups showed the same trend as those of PI3K (Fig. S5c and d). However, the H score of Bax was slightly increased in the control (25.92 ± 1.09) and TiO_2_ groups (19.56 ± 2.79), whereas the Bax H score significantly increased after FML, Tet, and Tet@TiO_2_ treatment, with the most obvious increase in the Tet@TiO_2_ group (58.81 ± 4.36) (Fig. S5e). We further analyzed the ratio of Bax/Bcl-2 in each group. This ratio was highest in the Tet@TiO_2_ group and lowest in the control and TiO_2_ groups, and there was no significant difference between the Bax/Bcl-2 ratio in the control and TiO_2_ groups (*P* = 0.439) (Fig. S5f). WB clearly revealed that the expression levels of PI3K, AKT, and Bcl-2 in the control and TiO_2_ groups were very similar to those in the normal group (Fig. S6a). However, the expression of these inflammatory factors decreased after treatment with FML, Tet, or Tet@TiO_2_. In addition, the expression of PI3K, AKT, and Bcl-2 in the Tet@TiO_2_ group was notably lower than that in the FML and Tet groups and even lower than that in the normal group (Fig. S6b to d). However, Bax was slightly up-regulated in the control and TiO_2_ groups compared with that in the normal group, but its expression increased significantly in the FML, Tet, and Tet@TiO_2_ groups, with the highest expression level in the Tet@TiO_2_ group (Fig. S6e). Additionally, the ratio of Bax/Bcl-2 was analyzed via WB, and the results were consistent with those determined by immunohistochemistry (Fig. S6f). In summary, Tet@TiO_2_ effectively down-regulated inflammatory factors (PI3K, AKT, and Bcl-2) and up-regulated Bax, thereby inhibiting the inflammatory PI3K–AKT–Bax/Bcl-2 signaling pathway and ultimately preventing the development of corneal haze in the third week after TransPRK. The change in the Bax/Bcl-2 ratio is highly important when evaluating the effect of a drug.

### RT-qPCR analysis of inflammatory factor mRNA expression after Tet@TiO_2_ treatment in vivo

Three weeks after TransPRK (and after 2 weeks of drug treatment), RNA was extracted from the corneal tissues of each group for RT-qPCR detection of the changes in PI3K, AKT, Bax, and Bcl-2 messenger RNA (mRNA) expression in the corneal tissues of each group; additionally, the changes in the Bax/Bcl-2 ratio were analyzed. Compared with those in the normal group, the relative mRNA levels of PI3K, AKT, and Bcl-2 genes were significantly greater in the control (PBS) and TiO_2_ groups. Furthermore, compared with those in the control and TiO_2_ groups, the relative mRNA levels of PI3K, AKT, and Bcl-2 in the FML, Tet, and Tet@TiO_2_ groups were significantly lower, whereas those in the Tet@TiO_2_ group were higher. However, compared with that in the normal group, the relative mRNA expression of Bax was slightly lower in the control and TiO_2_ groups, but Bax expression increased significantly in the FML, Tet, and Tet@TiO_2_ groups, with the most significant increase observed in the Tet@TiO_2_ group. Furthermore, the ratio of Bax/Bcl-2 in the FML, Tet, and Tet@TiO_2_ groups gradually increased, and this ratio was the greatest in the Tet@TiO_2_ group (Fig. S7).

### Detection of the antibacterial effects of Tet@TiO_2_ against MRSA, *E. coli*, and MRSE

As shown in Fig. S8, qualitative analysis showed that in the MRSA group, the average diameters of the LED, TDED, Tet@TiO_2_, and CED bacteriostatic rings were 3.78 ± 0.58, 3.02 ± 0.39, 3.08 ± 0.41, and 2.23 ± 0.37 cm, respectively. In the *E. coli* group, the average diameters of the LED, TDED, Tet@TiO_2_, and CED bacteriostatic rings were 2.54 ± 0.26, 2.43 ± 0.28, 3.12 ± 0.29, and 1.45 ± 0.29 cm, respectively. Furthermore, in the MRSE group, the average diameters of the LED, TDED, Tet@TiO_2_, and CED bacteriostatic rings were 4.22 ± 0.48, 3.13 ± 0.41, 3.15 ± 0.32, and 2.78 ± 0.29 cm, respectively. However, no clear bacteriostatic circles were observed after MRSA, *E. coli*, or MRSE were treated with FML. Together, these results showed that, qualitatively, Tet@TiO_2_ had good antibacterial performance (Figs. S9 to S11). Furthermore, LED, TDED, and CED produced their maximum bactericidal effects after 1 h in the order of LED > TDED > Tet@TiO_2_ > CED. However, after 2 and 4 h, the order changed to the following: Tet@TiO_2_ > LED > TDED > CED (Fig. S12), and this phenomenon was observed for MRSA, *E. coli*, and MRSE. Regardless of the type of bacteria, LED, TDED, Tet@TiO_2_, and CED showed the best antibacterial performance 1 h after administration. Although the initial antibacterial effects of LED and TDED were stronger than those of Tet@TiO_2_, their impact gradually weakened over time. Furthermore, Tet@TiO_2_ maintained strong antibacterial properties after 2 and 4 h that were better than those of LED and TDED because of the inherent characteristics of the nanomaterial.

## Discussion

Anti-inflammatory treatment is crucial for controlling the expression of inflammatory factors after refractive corneal surgery and preventing and treating corneal haze [38]. Many studies have reported that α-SMA, CTGF, and type III collagen play important roles in the development of corneal haze, and the symptoms of this disease can be significantly alleviated by inhibiting the expression of those cytokines [39–41]. Traditional anti-inflammatory agents such as corticosteroids can cause serious complications, including intraocular hypertension and cataracts [11,12], which limit their use in corneal haze treatment. In recent years, nanomaterials have been used as drug carriers to construct delivery systems with good sustained release properties, targeting abilities and biocompatibility, thus successfully avoiding the complications of corticosteroids. Therefore, it is worth developing new multifunctional synthetic nanodrugs with anti-inflammatory, antibacterial, slow drug release, and targeting properties and low toxicity to prevent and treat haze after refractive corneal surgery.

First, we examined the physicochemical properties of Tet@TiO_2_ immediately after its synthesis. Collectively, the diameter and zeta potential measurements, morphological observations, and UV absorption spectrum of Tet@TiO_2_ revealed that Tet was successfully loaded onto TiO_2_. Additionally, the average size of Tet@TiO_2_ measured over 7 d demonstrated its good stability. The drug release curves constructed from data at different temperatures revealed that Tet@TiO_2_ was stable at 4 and 25 °C, but the drug was released at 33 °C. In addition, the EE and LC of Tet further indicated the successful synthesis of this new nanomedicine. The cornea is mainly divided into the epithelial layer, anterior elastic layer, stromal layer, posterior elastic layer, and endothelial layer. The corneal epithelial layer is located at the outermost layer of the cornea and is a nonkeratinized squamous epithelial cell with 5 to 6 layers, approximately 35 μm thick, and neatly arranged. The anterior corneal elastic layer, also known as the Bowman layer, is composed of nonoriented collagen fibers and glucosamine. It is 8 to 14 μm thick and has no cells. After birth, it cannot regenerate due to damage. The ophthalmic branch of the trigeminal nerve sends out 60 to 80 peripheral fibers around the cornea and demyelinates them. It first travels in the shallow stromal layer and forms a dense nerve plexus below the anterior elastic layer. It passes through many fine tubules of the anterior elastic layer and epithelial basement membrane to reach the surface of the epithelial layer. Our previous studies have shown that controlling the size of nanoparticles between 80 and 150 nm can effectively penetrate the intercellular spaces of eye tissues, increase the penetration of drugs into the eye, and improve treatment efficacy. Therefore, we believe that particles with a size of about 100 nm have the best size in this study.

Next, we explored the targeting abilities of Tet@TiO_2_ in vivo and in vitro. In the cell experiments, TGF-β_2_ (4 ng/ml) was administered to construct a cell model with characteristics similar to those of corneal haze. The results demonstrated that inflamed HCSFs had an outstanding ability to take up Tet@TiO_2_, whereas normal HCSFs did not exhibit obvious Tet@TiO_2_ uptake. More importantly, DiI-labeled Tet@TiO_2_ was used to treat corneas in vivo for 0.5, 1, and 2 h on the fifth day after TransPRK. Over time, the DiI fluorescence signal gradually penetrated the stromal layer from the corneal epithelium in the corneal haze animal model to generate a diffusely distributed fluorescence signal in the stromal layer of the cornea. In the normal cornea, the DiI fluorescence signal appeared only on the corneal surface, and there was almost no DiI fluorescence was detected in the stroma. The above results showed that, compared with normal model eyes, the corneal haze inflammation model eyes more easily took up Tet@TiO_2_. In other words, Tet@TiO_2_ effectively targeted corneal haze both in vivo and in vitro.

Additionally, we tested the biosafety of FML, Tet, and Tet@TiO_2_ using CCK-8, FCM, and live/dead staining assays. The HCSFs showed good viability after treatment with even a very high concentration of Tet@TiO_2_ (40 μg/ml). Furthermore, at 4 μg/ml, FML, Tet, and Tet@TiO_2_ were safe for HCSFs overall, and the percentage of living cells in the Tet@TiO_2_ group was greater than that in the FML and Tet groups after 24 h of incubation. In addition, live/dead staining revealed that the number of dead cells in the Tet@TiO_2_ group was significantly lower than that in the FML and Tet groups after 24 h. To examine in vivo biosafety, Tet@TiO_2_ eye drops were administered to New Zealand white rabbits that had not undergone surgery 3 times a day for 30 d, and no significant redness or increase in secretion was detected. In addition, corneal opacity, corneal epithelial damage, and corneal neovascularization were not observed. Prolonged use of FML can easily lead to increased intraocular pressure. Notably, Tet@TiO_2_ did not increase intraocular pressure but rather reduced it. Moreover, rabbit blood was collected for further analyses of routine blood indices and liver and kidney function indicators, among other factors. These results showed that Tet@TiO_2_ did not affect the WBC, LYM, or PLT counts; the HGB, ALT, AST, Crea, or BUN levels; or the eGFR in the experimental animals. Furthermore, the major organs, such as the heart, liver, spleen, lungs, and kidneys, were stained with H&E, and no significant abnormalities were observed, confirming the good biocompatibility of Tet@TiO_2_. Moreover, we found that the corneal epithelium in the FML group repeatedly presented diffuse, punctate fluorescence staining and that the Tet group presented punctate staining, but fluorescence staining was no longer observed in the Tet@TiO_2_ group after 2 weeks of treatment. Collectively, these results indicated that Tet@TiO_2_ had good biocompatibility both in vivo and in vitro.

To determine the therapeutic effect of Tet@TiO_2_ on corneal haze after refractive corneal surgery, we analyzed the clinical and pathological manifestations of rabbit corneal haze at different time points after TransPRK. The clinical manifestations of corneal haze in the Tet@TiO_2_ group basically disappeared, whereas the clinical manifestations of corneal haze were still noticeable in the FML and Tet groups after 2 weeks of treatment. Then, we used H&E staining, polarized light microscopy and TEM to observe the changes in the macro- and microstructures of the corneas in each group. H&E staining revealed that the corneal epithelium and stroma in the FML and Tet groups still presented different degrees of edema and that the arrangement of the collagen fibers was still disordered. However, in the Tet@TiO_2_ group, the edema in the corneal epithelium and stromal layer was significantly reduced, basal cell vacuolar degeneration cells disappeared, and the shape of the corneas returned to almost normal with regularly arranged collagen fibers. In addition, Sirius red staining revealed that the area positive for type I and III collagen fibers in the Tet@TiO_2_ group was significantly smaller than that in the FML and Tet groups. Additionally, TEM revealed the presence of many fibroblasts in the stromal layers of the surgical areas in the control and TiO_2_ groups; the cell volume and nuclei were enlarged; the extracellular collagen fibers were uneven and disorderly arranged; the cells were rich in rough endoplasmic reticula, mitochondria, Golgi apparatuses, and other organelles; and the basement membranes were thin and discontinuous. These results indicated the high metabolism in and vigorous proliferation of the control and TiO_2_ cells. In the FML and Tet groups, the extracellular collagen fibers were uneven in thickness and disordered in arrangement, and there was an abundance of rough endoplasmic reticula, mitochondria, Golgi apparatuses, and other organelles. However, the extracellular collagen fibers in the Tet@TiO_2_ group were uniform in thickness and regular in arrangement, and few fibroblasts were noted in the anterior stromal layer. In addition, the cell and nucleus volumes in the Tet@TiO_2_ group were significantly smaller than those in the other groups, and the contents of organelles were lower, suggesting that the metabolism and proliferation of the cells in this group were low. Three weeks after TransPRK, corneal tissue was extracted from each group for proteomic analysis, and the results revealed that α-SMA, CTGF, and type III collagen were significantly up-regulated in the control and TiO_2_ groups. However, compared with those in the FML and Tet groups, the down-regulation of α-SMA, CTGF, and type III collagen in the Tet@TiO_2_ group was more obvious after treatment for the same length of time. In summary, Tet@TiO_2_ can effectively prevent and treat corneal haze after refractive corneal surgery.

Immunohistochemical analysis showed that the PI3K, AKT, and Bcl-2 expression intensity in the control group was level 3 at 3 weeks after TransPRK, with strongly positive, brown corneal tissue; the Bax expression intensity was level 1, with pale, yellow and weakly positive corneal tissue. However, the expression intensities of PI3K, AKT, and Bcl-2 significantly decreased after Tet@TiO_2_ treatment and that of Bax significantly increased, and the ratio of Bax/Bcl-2 was highest in the Tet@TiO_2_ group. Additionally, Western blot analysis of the corneal tissue from each group revealed trends in PI3K, AKT, Bcl-2, and Bax expression that were consistent with the immunohistochemical results. These data suggest that Tet@TiO_2_ can effectively down-regulate the inflammatory factors PI3K, AKT, and Bcl-2 and up-regulate Bax, thereby inhibiting the inflammatory PI3K–AKT–Bax/Bcl-2 signaling pathway and thus reducing the expression and release of α-SMA, CTGF, and type III collagen to ultimately prevent and treat corneal haze. The above results were further confirmed via RT-qPCR. Research has shown that the intracellular PI3K–AKT signal transduction pathway plays key roles in maintaining the normal physiological functions of cells, such as growth, differentiation, and metabolism [42,43]. In addition, apoptosis, gene transcription, protein translation, metabolism, angiogenesis, and the cell cycle are regulated through this pathway. Activated PI3K can phosphorylate phosphoinositol to activate AKT activation via the phosphoinositol-dependent kinase-1-mediated phosphorylation of serine 308 and threonine 473. Moreover, both phosphatidylinositol 4,5-diphosphate (PIP2) and PIP3 act as secondary messengers to transmit intracellular signals to activate the serine/threonine protein kinase AKT. Under the synergistic action of phosphatidylinositol-dependent protein kinase, PIP2 and PIP3 combine with AKT, which leads to AKT translocation from the cytoplasm to the cell membrane, thus promoting AKT phosphorylation. Activated PI3K–AKT can regulate apoptosis by phosphorylating target proteins in a variety of downstream pathways [44,45]. Among them, the Bcl-2 gene family plays a key role in apoptosis regulation. Reports have shown that Bcl-2 is an antiapoptotic factor that does not affect cell proliferation but instead prevents apoptosis upon the application of various physiological and pathological stimuli. In addition, Bax is a member of the Bcl-2 gene family that promotes apoptosis. Bcl-2 and Bax usually exist as dimers. Since Bcl-2 inhibits apoptosis but Bax promotes apoptosis, the ratio of Bax/Bcl-2 regulates cell apoptosis and survival [46,47]. In this study, the PI3K–AKT signaling pathway was activated after refractive corneal surgery, which led to the up-regulation of the antiapoptotic protein Bcl-2, which further promoted the development of corneal haze. However, Tet@TiO_2_ reduced AKT phosphorylation by inhibiting PI3K expression, thus blocking PI3K–AKT signal transmission and effectively up-regulating the proapoptotic protein Bax, which led to Bcl-2 down-regulation and increased corneal myofibroblast apoptosis. The expression of inflammatory factors involved in the development of corneal haze, such as α-SMA, CTGF, and type III collagen, decreased after Tet@TiO_2_ treatment because it suppressed PI3K–AKT–Bcl-2 signaling. Thus, fewer organelles, such as rough endoplasmic reticula, mitochondria, and Golgi apparatuses, were found in the cells in the surgical area; the fibroblasts in the corneal stroma displayed weakened activation and proliferative activities; and ECM accumulation decreased. Together, these changes can hinder the development of corneal haze. In summary, we believe that Tet@TiO_2_ can inhibit corneal haze development by regulating PI3K–AKT–Bax/Bcl-2 signaling after refractive corneal surgery. The change in the Bax/Bcl-2 ratio is highly important when evaluating the effect of a drug.

Titanium dioxide, which has photocatalytic, antibacterial, and corrosion resistance properties and degrades naturally, is widely used in orthopedic and dental implant materials [14,48] and has emerged as a potential material for ophthalmic applications in recent years [16,17]. In terms of antibacterial activity, titanium dioxide has been shown to produce photogenerated electron–hole pairs under natural light irradiation and further produce highly oxidizing reactive oxygen species, which react with the organic components of bacteria to disrupt their physiological balance and ultimately kill them [49]. In this study, we chose MRSA, *E. coli*, and MRSE as the research objects and compared the antibacterial properties of Tet@TiO_2_, FML, LED, TDED, and CED by qualitative and quantitative methods. We found that the antibacterial properties of Tet@TiO_2_ were not as strong as those of LED and TDED soon after drug application, but they were stronger than those of CED. With increasing time, the antibacterial effects of LED, TDED, and CED gradually decreased, but the high activity of Tet@TiO_2_ was maintained because of the inherent characteristics of the nanomaterial. The above results indicate that the antibacterial performance of titanium dioxide is worthy of further exploration for ophthalmological applications.

In conclusion, we used mesoporous titanium dioxide as a carrier and attached Tet to its mesopores to synthesize a new nanodrug, Tet@TiO_2_, in this study. Compared with FML and Tet, Tet@TiO_2_ showed controlled drug release, good inflammation-targeting behavior, and improved biocompatibility both in vivo in a corneal haze model and in vitro in inflammatory HCSFs. After refractive corneal surgery, Tet@TiO_2_ can modulate inflammatory PI3K–AKT–Bax/Bcl-2 signaling in rabbit corneas by down-regulating PI3K, AKT, and Bcl-2 and up-regulating Bax. This leads to inhibition of the expression and release of inflammatory factors, such as α-SMA, CTGF, and type III collagen, to ultimately prevent and treat corneal haze. In addition, we discovered that Tet@TiO_2_ has robust antibacterial performance and improved safety. Therefore, this study provides a new strategy to explore drugs with both anti-inflammatory and antibacterial properties. Our next research goal is determining how to better apply this drug in the clinic.

## Ethical Approval

This study adhered to the tenets of the Declaration of Helsinki and Malaysian Guidelines for Good Clinical Practice and the ARRIVE guidelines. This study protocol was reviewed and approved by the Ethics Committee of Chongqing Medical University (no. 20210231).

## Data Availability

The datasets used and/or analyzed during the current study are available from the corresponding author on reasonable request. All data generated or analyzed during this study are included in this published article.
