# Supplementary figures and images for: Multifunctional Mesoporous Titanium Dioxide Nanodrug for Corneal Haze Treatment and Its Mechanism
Source: Biomater Res. 2025 May 14;29:0202. doi: 10.34133/bmr.0202 (PMC12076154; doi:10.34133/bmr.0202)

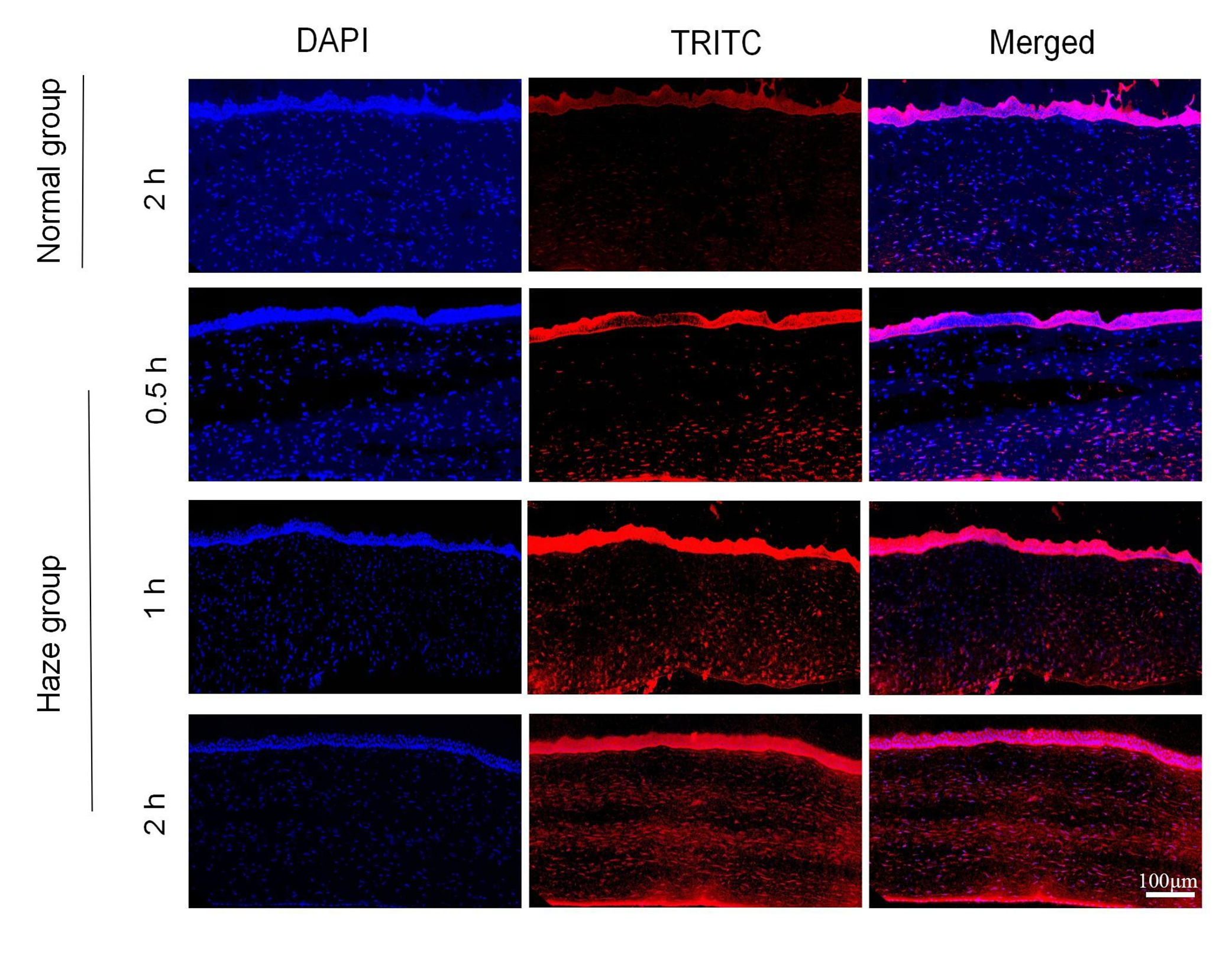

Supplement: Supplementary 1 — Figs. S1 to S12 [file bmr.0202.f1.zip › Fig. S1.tif]

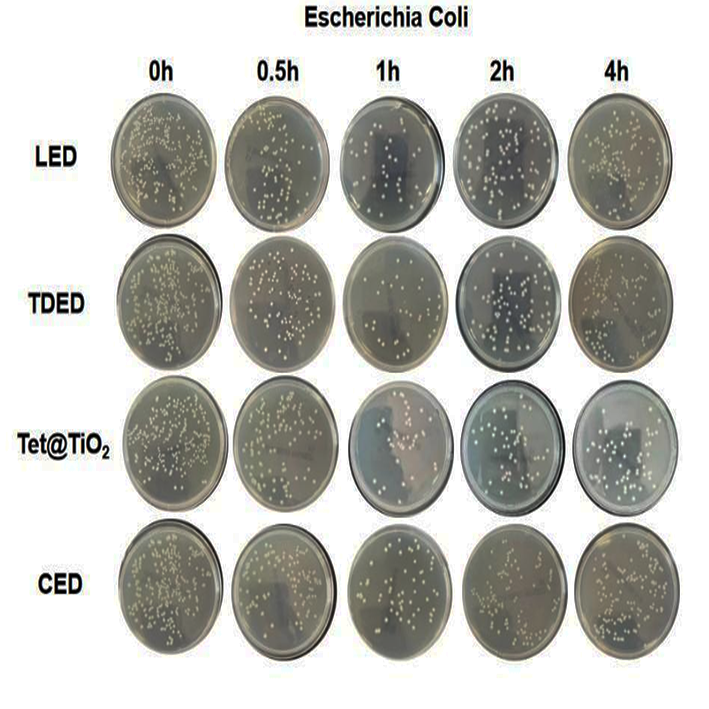

Supplement: Supplementary 1 — Figs. S1 to S12 [file bmr.0202.f1.zip › Fig. S10.tif]

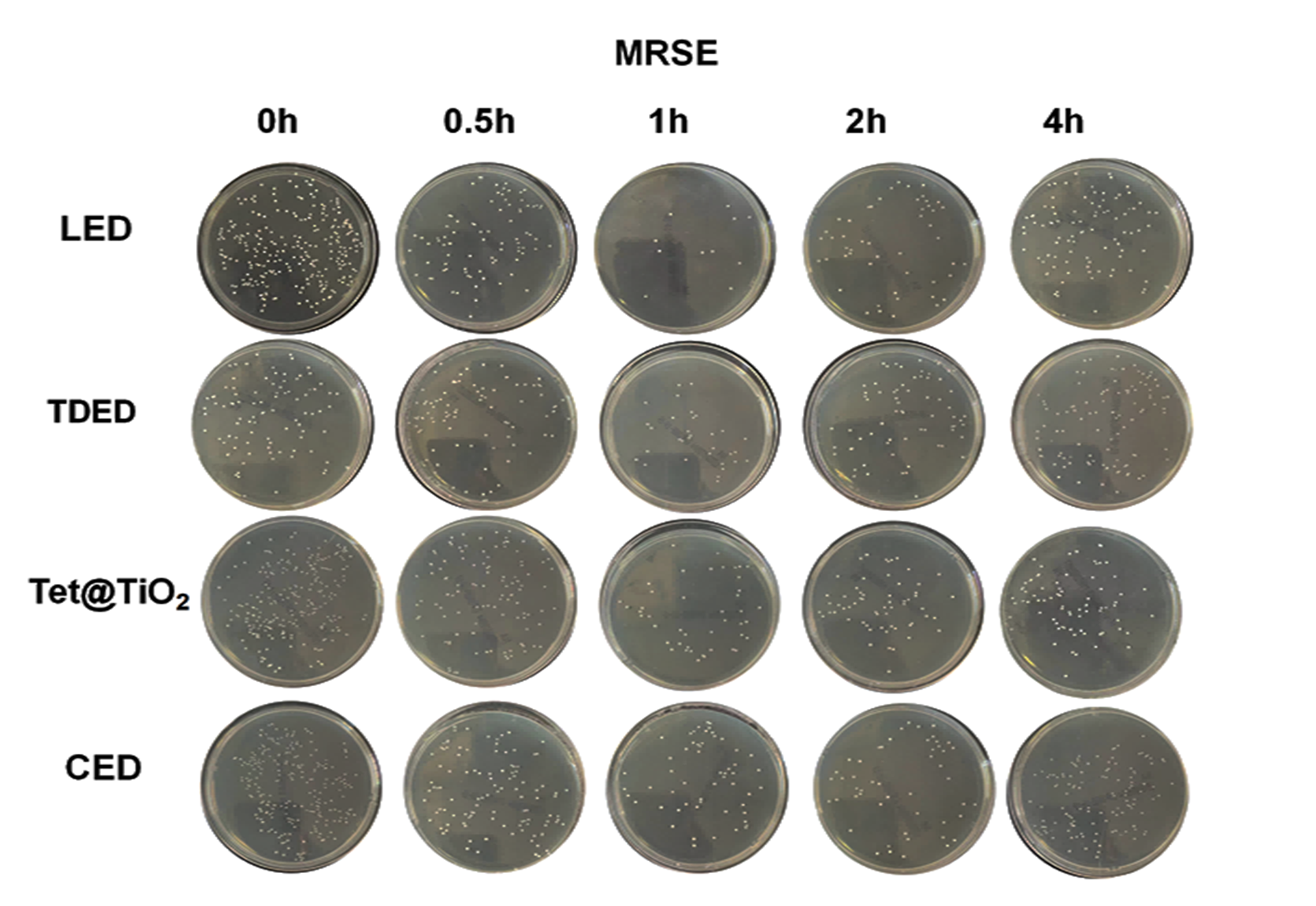

Supplement: Supplementary 1 — Figs. S1 to S12 [file bmr.0202.f1.zip › Fig. S11.tif]

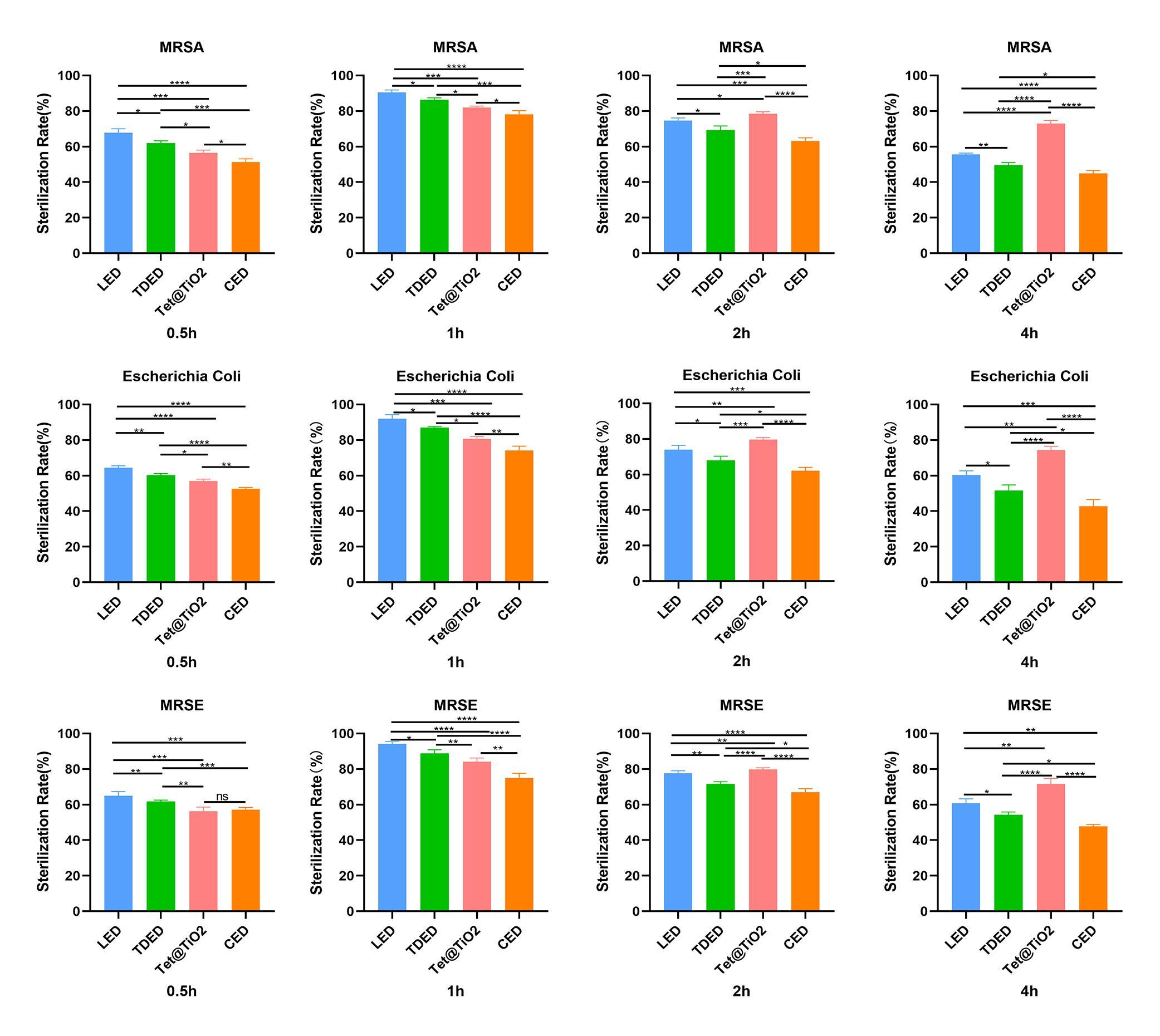

Supplement: Supplementary 1 — Figs. S1 to S12 [file bmr.0202.f1.zip › Fig. S12.tif]

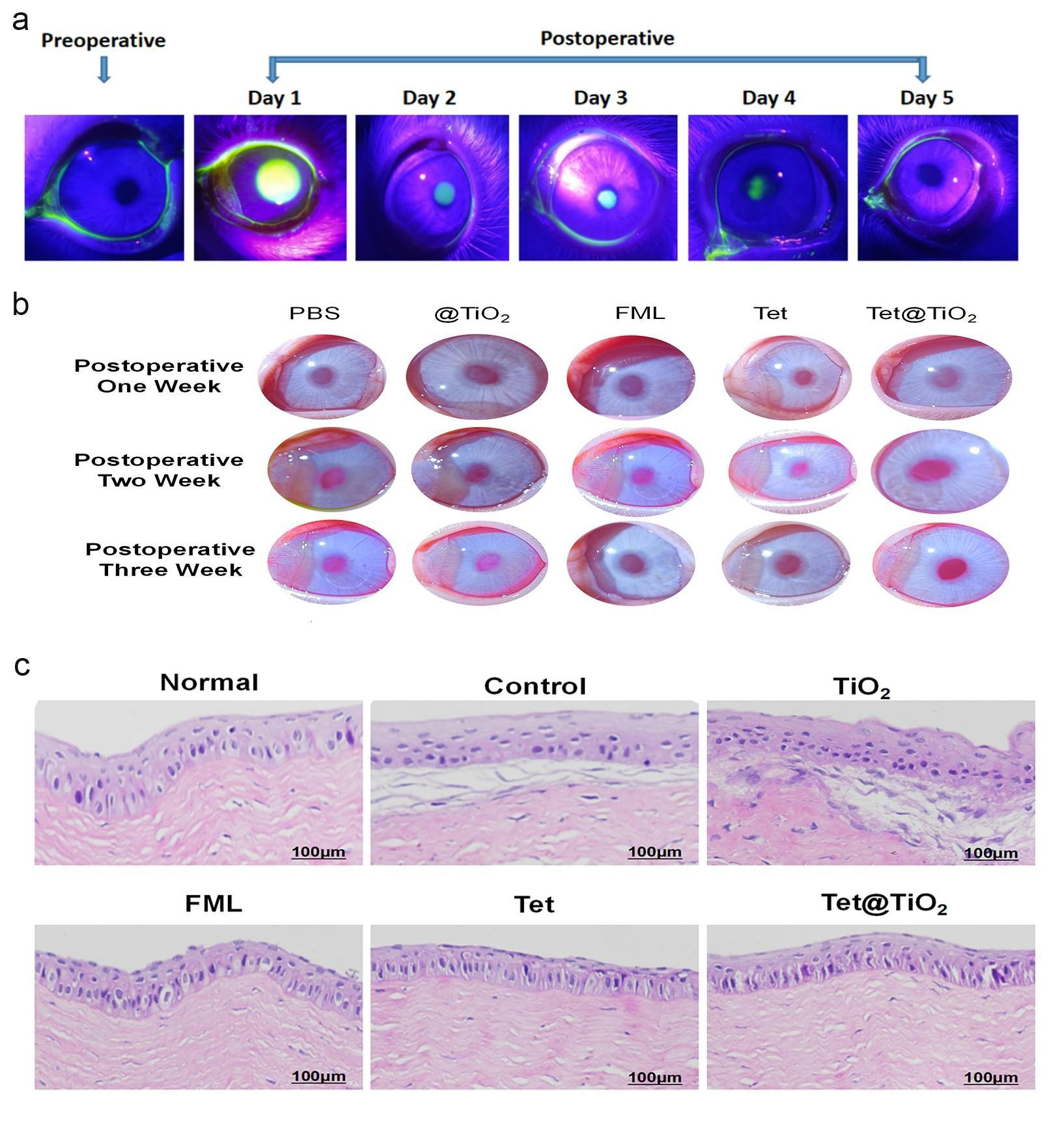

Supplement: Supplementary 1 — Figs. S1 to S12 [file bmr.0202.f1.zip › Fig. S2.tif]

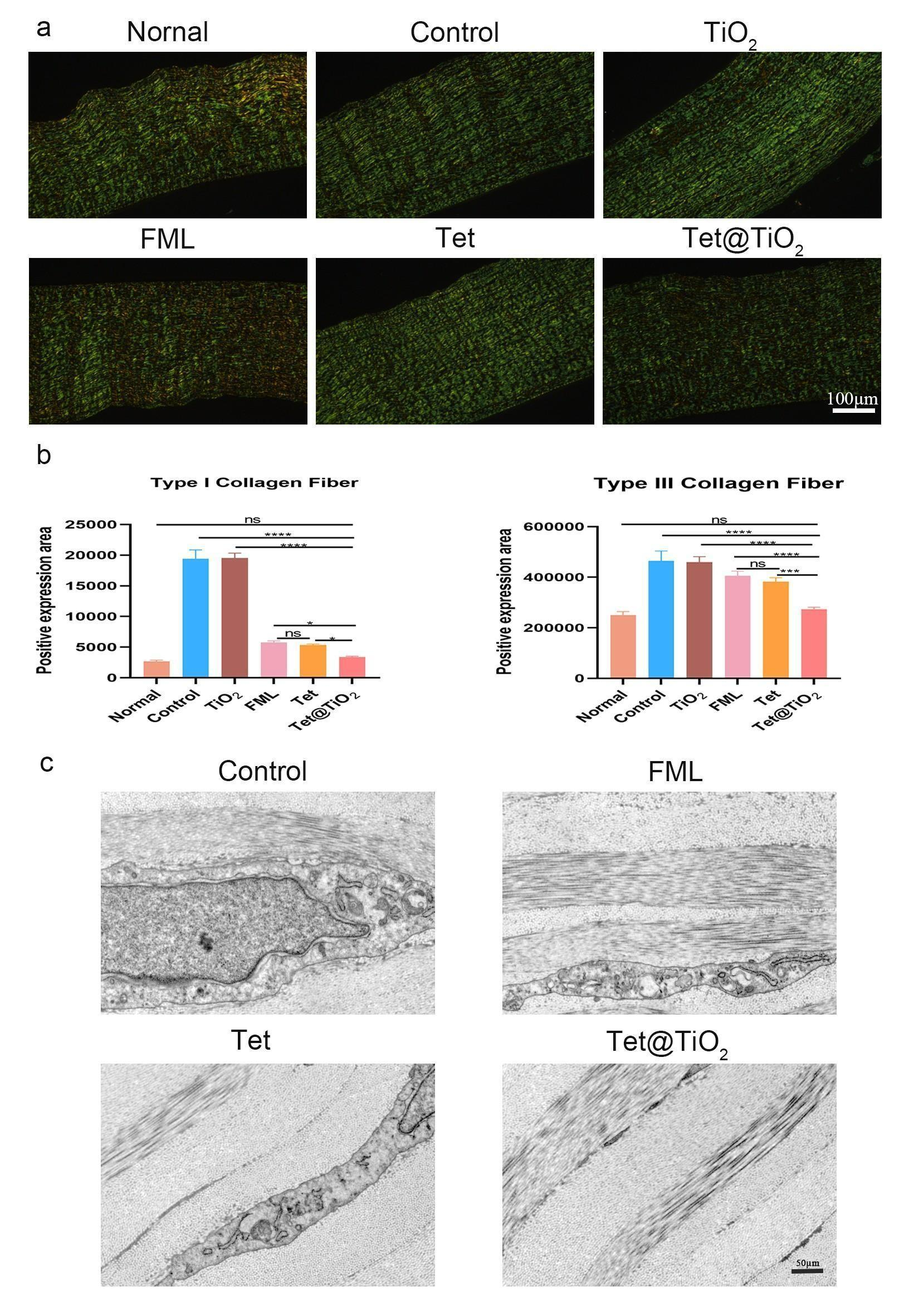

Supplement: Supplementary 1 — Figs. S1 to S12 [file bmr.0202.f1.zip › Fig. S3.tif]

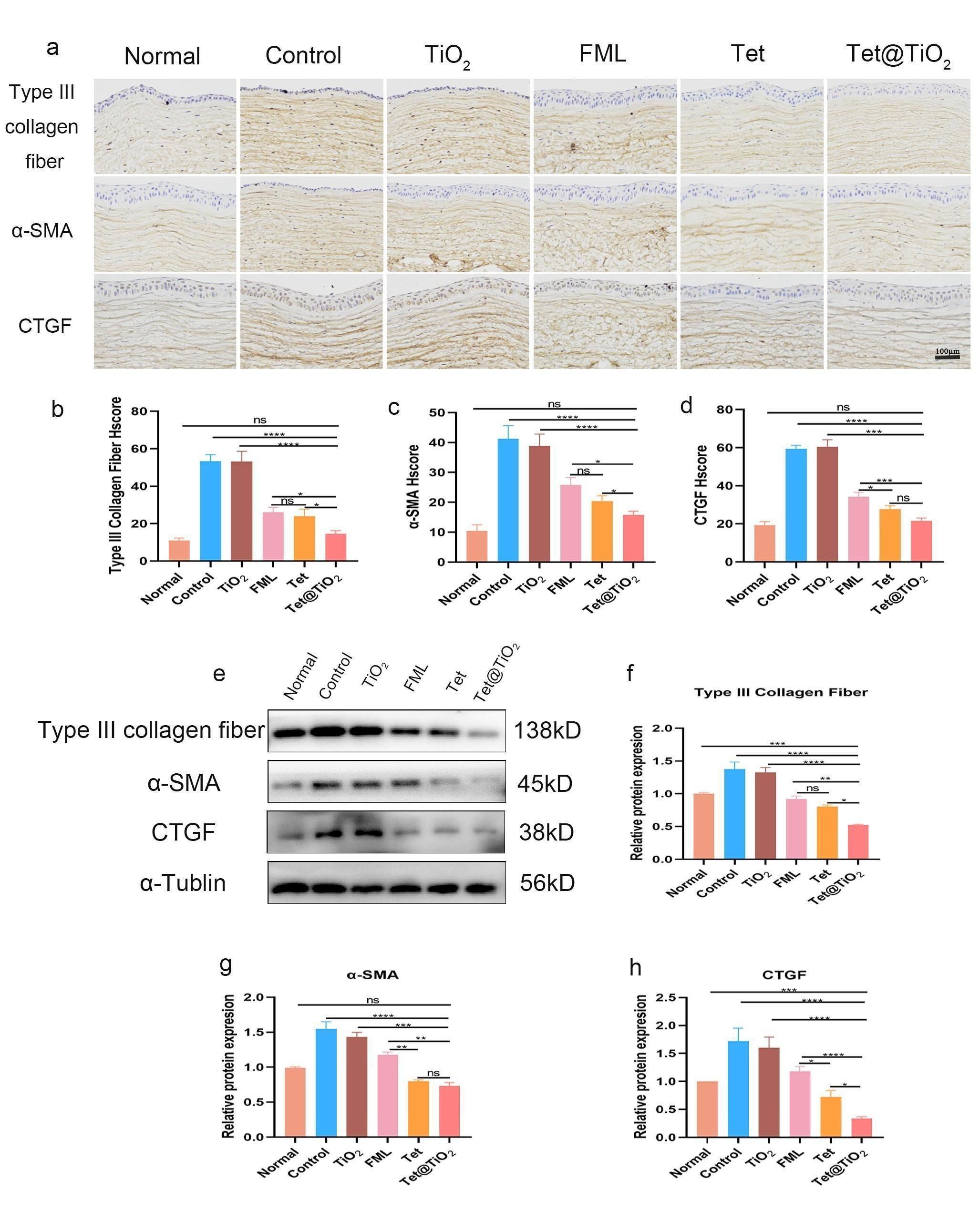

Supplement: Supplementary 1 — Figs. S1 to S12 [file bmr.0202.f1.zip › Fig. S4.tif]

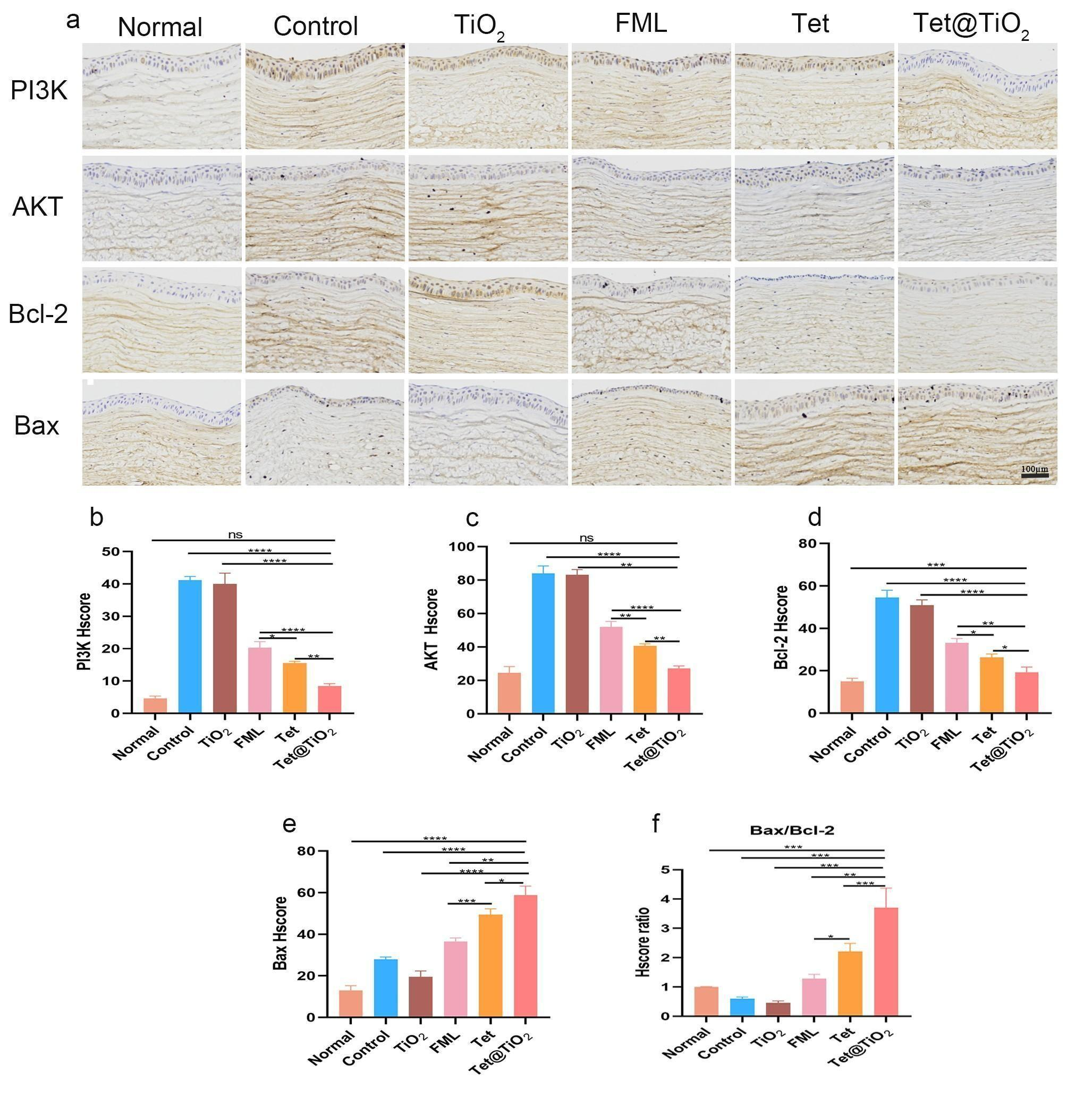

Supplement: Supplementary 1 — Figs. S1 to S12 [file bmr.0202.f1.zip › Fig. S5.tif]

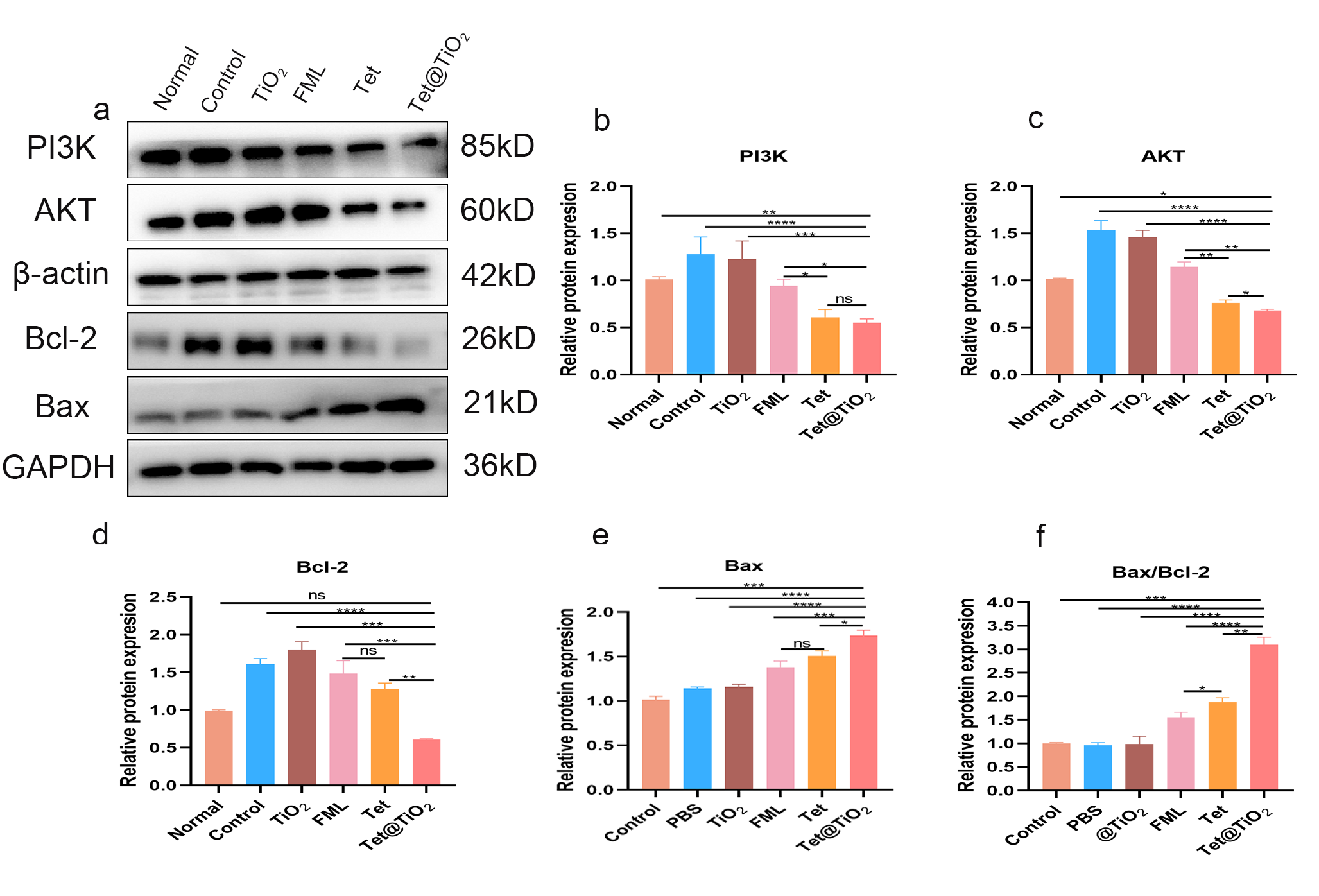

Supplement: Supplementary 1 — Figs. S1 to S12 [file bmr.0202.f1.zip › Fig. S6.tif]

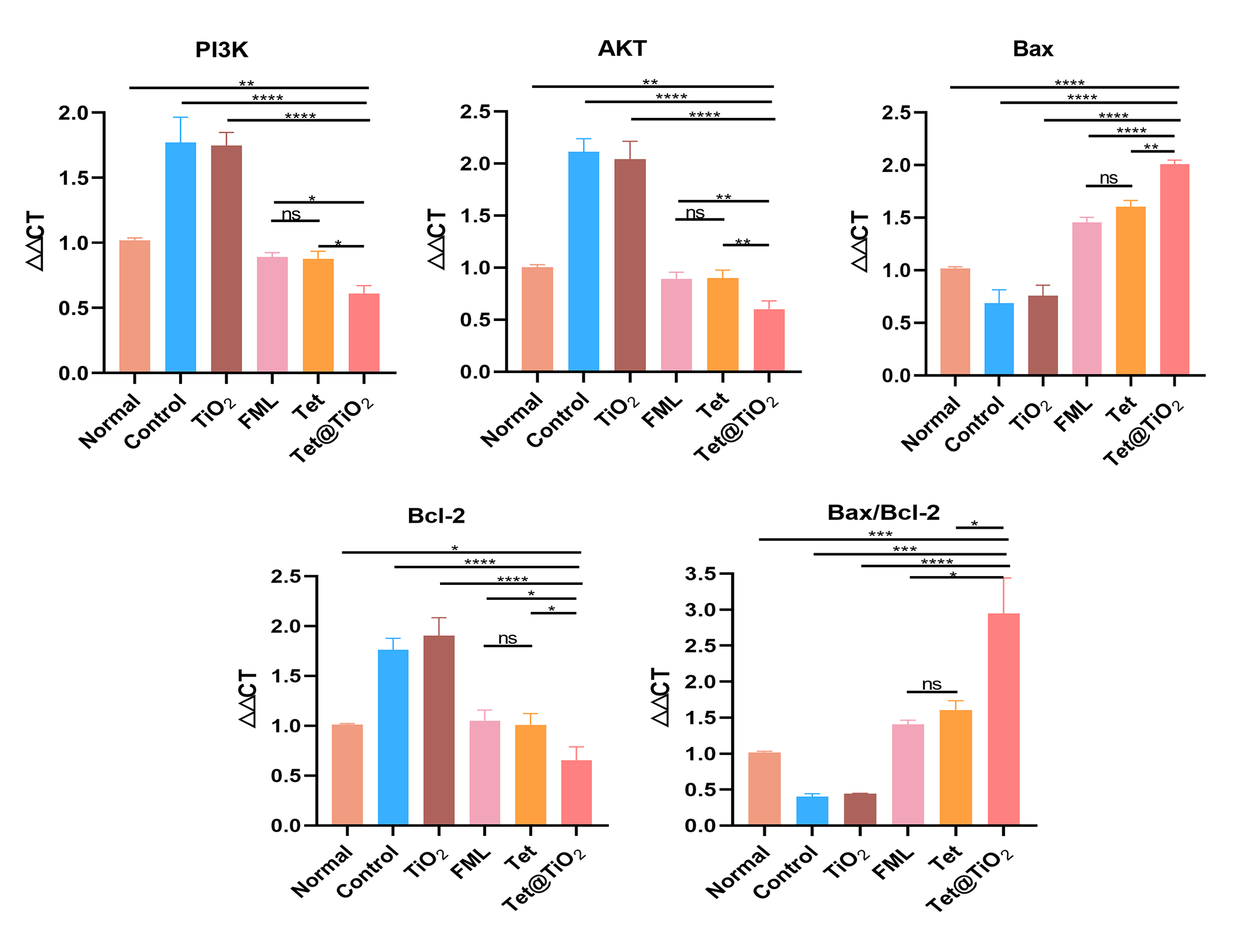

Supplement: Supplementary 1 — Figs. S1 to S12 [file bmr.0202.f1.zip › Fig. S7.tif]

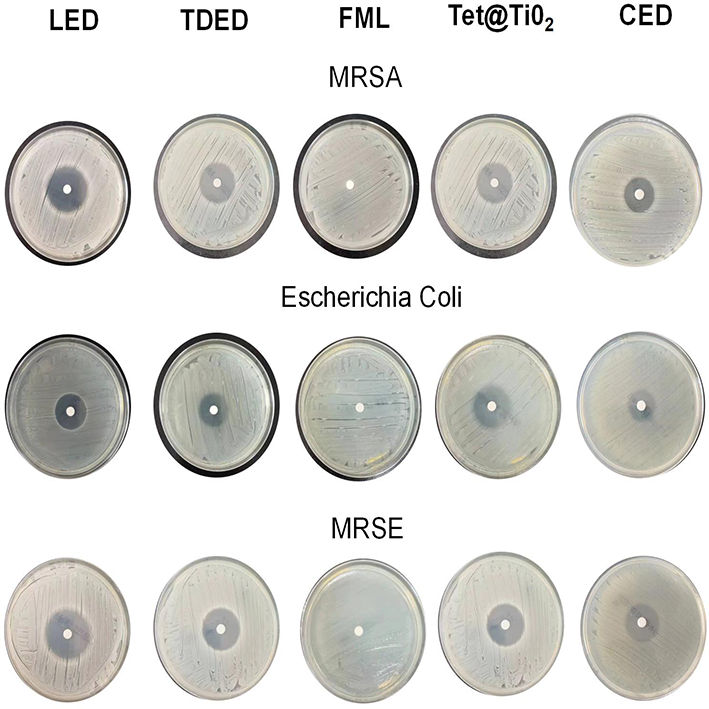

Supplement: Supplementary 1 — Figs. S1 to S12 [file bmr.0202.f1.zip › Fig. S8.tif]

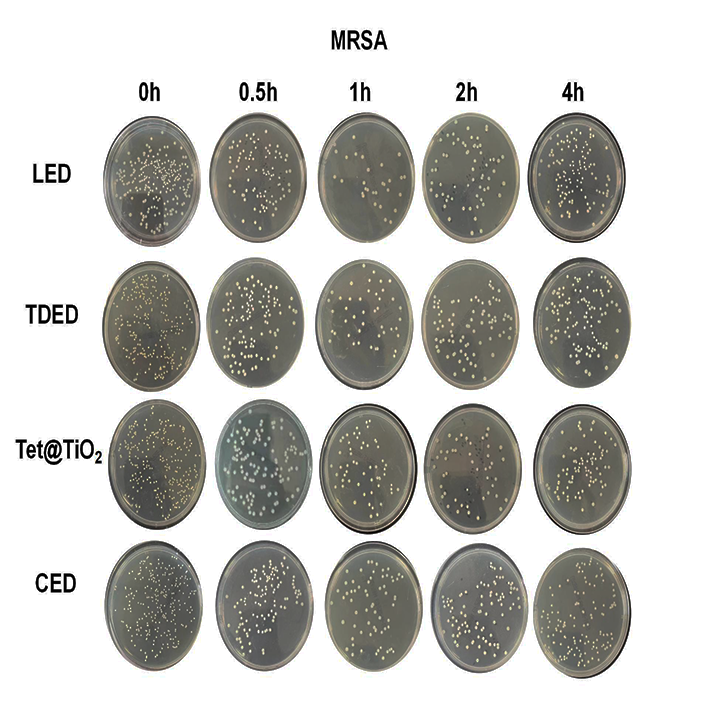

Supplement: Supplementary 1 — Figs. S1 to S12 [file bmr.0202.f1.zip › Fig. S9.tif]
