# Supplementary material for: Multifunctional Mesoporous Titanium Dioxide Nanodrug for Corneal Haze Treatment and Its Mechanism
Source: Biomater Res. 2025 May 14;29:0202. doi: 10.34133/bmr.0202 (PMC12076154; doi:10.34133/bmr.0202)
Supplement: Supplementary 1 — Figs. S1 to S12 [file bmr.0202.f1.zip › sm.doc]

**Fig. S1.**Fluorescence images of DiI-labeled Tet@TiO_2_ (1 mg/mL) in the corneal stroma of normal and haze model rabbit eyes. Blue represents nuclei stained with DAPI under UV excitation, and red represents the DiI-labeled Tet@TiO_2_ signal in the corneal stroma.

**Fig. S2.**. a) Fluorescence staining images of rabbit corneas before and 1–5 days after surgery. b) Changes in corneal haze in each group one, two and three weeks after TransPRK. c). H&E staining images of the corneas of rabbits in the experimental and control groups 3 weeks after TransPRK.

**Fig. S3.**. a) Areas with positive type I and III collagen staining in each group observed with an Eclipse Ci-L polarized light microscope at 3 weeks after TransPRK. b) Numbers of type I and III collagen fibers determined by Image-Pro Plus 6.0 software (*P<0.05, **P<0.01, ***P<0.001, ****P<0.0001). c) Ultrastructural changes in corneal tissue in each group were observed via TEM 3 weeks after TransPRK.

**Fig. S4**. a) The intensities of α-SMA, CTGF and type III collagen expression in each group were determined via immunohistochemistry at 3 weeks after TransPRK. b, c, d)Immunohistochemical analysis of α-SMA, CTGF and type III collagen expression in corneal tissue. e) The expression levels of α-SMA, CTGF and type III collagen in each group determined via WB at 3 weeks after TransPRK. f, g, h) Quantification of α-SMA, CTGF and type III collagen expression in corneal tissue via WB. Ordinary one-way ANOVA; *P<0.05, **P<0.01, ***P<0.001, ****P<0.0001.

**Fig. S5**. a) Intensities of PI3K, AKT, Bcl-2 and Bax expression in the PI3K-AKT-Bax/Bcl-2 signaling pathway in each group determined via immunohistochemistry in the 3rd week after TransPRK. b, c, d, e, f)Immunohistochemical determination of PI3K, AKT, Bcl-2 and Bax expression and the Bax/Bcl-2 ratio in corneal tissue. Ordinary one-way ANOVA; *P<0.05, **P<0.01, ***P<0.001, ****P<0.0001.

**Fig. S6**. a) Expression of PI3K, AKT, Bcl-2, and Bax in the PI3K-AKT-Bax/Bcl-2 signaling pathway in each group determined via WB in the 3rd week after TransPRK. b, c, d, e, f) WB analysis of PI3K, AKT, Bcl-2, Bax, and Bax/Bcl-2 expression in corneal tissue. Ordinary one-way ANOVA; *P<0.05, **P<0.01, ***P<0.001, ****P<0.0001.

**Fig. S7**. RT‒qPCR analysis of PI3K, AKT, Bax, and Bcl-2 mRNA expression in corneal tissue. Ordinary one-way ANOVA; *P<0.05, **P<0.01, ***P<0.001, ****P<0.0001.

**Fig. S8**. Qualitative assessment of the antibacterial effects of LED, TDED, FML and Tet@TiO_2_.

**Fig. S9**. Changes in the quantity of visible MRSA colonies in the LED, TDED, Tet@TiO_2_ and CED groups before and after treatment for different durations, as determined by the plate count method.

**Fig. S10**. Changes in the quantity of visible *Escherichia coli* colonies in the LED, TDED, Tet@TiO_2_ and CED groups before and after treatment for different durations, as determined by the plate count method.

**Fig. S11**. Changes in the quantity of visible MRSE colonies in the LED, TDED, Tet@TiO_2_ and CED groups before and after treatment for different durations, as determined by the plate count method.

**Fig. S12**. a, b, c) Rates of MRSA, *Escherichia coli* and MRSE sterilization in the LED, TDED, Tet@TiO_2_ and CED groups after different durations. Ordinary one-way ANOVA; *P<0.05, **P<0.01, ***P<0.001, ****P<0.0001.
